# Structural modification of octadecanoic acid-3,4-tetrahydrofuran diester and the acaricidal activity and mechanism of its derivatives against *Sarcoptes* *scabiei* var. *Cuniculi*

**DOI:** 10.3389/fphar.2022.953284

**Published:** 2022-08-22

**Authors:** Lixia Li, Yu Zhang, Tao Liu, Rui Xing, Shuwei Peng, Xu Song, Yuanfeng Zou, Xinghong Zhao, Renyong Jia, Hongping Wan, Lizi Yin, Gang Ye, Fei Shi, Yingying Zhang, Guizhou Yue, Zhongqiong Yin

**Affiliations:** ^1^ Natural Medicine Research Center, College of Veterinary Medicine, Sichuan Agricultural University, Chengdu, China; ^2^ Key Laboratory of Animal Disease and Human Health of Sichuan Province, Sichuan Agricultural University, Chengdu, China; ^3^ College of Animal Science and Technology, Sichuan Agricultural University, Chengdu, China; ^4^ College of Science, Sichuan Agricultural University, Ya'an, China

**Keywords:** octadecanoic acid-3,4-tetrahydrofuran diester, benzoic acid-2-benzyloxy-3,4tetrahydrofuran diester, *S. scabiei* var. *cuniculi*, transcriptomics, energy metabolism

## Abstract

Octadecanoic acid-3,4-tetrahydrofuran diester is a compound with acaricidal activity isolated and extracted from neem oil. In this study, a series of derivatives were obtained by structural modification of octadecanoic acid-3,4-tetrahydrofuran diester. The acaricidal activity of these derivatives indicated that introduction of benzyloxy substitution at the 2-position of the furan ring and the formation of a benzoate at the 3,4-position of the furan ring (benzoic acid-2-benzyloxy-3,4-tetrahydrofuran diester) could enhance the acaricidal activity. At concentration of 20, 10, and 5 mg/ml, the median lethal time (LT_50_) values of benzoic acid-2-benzyloxy-3,4-tetrahydrofuran diester were 16.138, 47.274, and 108.122 min, respectively. The LC_50_ value of benzoic acid-2-benzyloxy-3,4-tetrahydrofuran diester at 60 min was 5.342 mg/ml. Transmission electron microscopy showed that after treatment with benzoic acid-2-benzyloxy-3,4-tetrahydrofuran diester, the body structure of mites was destroyed; dermal organelles were dissolved; nuclear chromatin was ablated. Further, transcriptome sequencing analysis was used to get insight into the acaricidal mechanism of benzoic acid-2-benzyloxy-3,4-tetrahydrofuran diester. The results showed that its acaricidal mechanism is related to interfering “energy metabolism” in *S. scabiei*, including processes such as citric acid cycle, oxidative phosphorylation pathway and fatty acid metabolism. Additionally, through the activity detection of the mitochondrial complexes of *S. scabiei*, it was further verified that the acaricidal mechanism of benzoic acid-2-benzyloxy-3,4-tetrahydrofuran diester was related to the energy metabolism system of *S. scabiei*.

## 1 Introduction

*Sarcoptes scabiei* var. *cuniculi* (*S. scabiei*) is a pathogen that can cause infections on the skin surface, which will reduce the quality of animal products and even lead to the death of animals (Seddiek et al., 2013; D’Ovidio and Santoro, 2021). In our previous study, a new compound octadecanoic acid-3,4-tetrahydrofuran diester with potent acaricidal activity was isolated from neem oil (Du et al., 2008, 2009; Deng et al., 2012; Chen et al., 2014; Song et al., 2017). However, this compound has disadvantages such as low extraction efficiency, poor solubility, difficult synthesis and low synthesis yield (Du et al., 2009; Deng et al., 2012). In order to improve the acaricidal activity of octadecanoic acid-3,4-tetrahydrofuran diester, in this study, its chemical structure was modified to obtain a series of octadecanoic acid-3,4-tetrahydrofuran diester derivatives. By testing the acaricidal activity, it was aimed to screen out the compounds with good acaricidal activity, and to preliminarily understand their structure-activity relationship. Transcriptiome sequencing is a powerful tool for analyzing gene expression changes in response to various environmental stresses. Based on the preliminary knowledge of the structure-activity relationship of the derivatives of octadecanoic acid-3,4-tetrahydrofuran diester, we further explored the mechanism and target of its acaricidal activity through RNA sequencing and transcriptional profiling analysis. It can provide a theoretical basis for the comprehensive development and utilization of octadecanoate-3,4-tetrahydrofuran diester derivatives.

## 2 Materials and methods

### 2.1 Materials

Octadecanoic acid-3,4-tetrahydrofuran diester was obtained from the chloroform extract of neem (Azadirachta indica) oil through re-crystallization in acetone (Du et al., 2009). Octadecanoic acid-3,4-tetrahydrofuran diester derivatives were synthesized by College of Science, Sichuan Agricultural University. Liquid paraffin was supplied by Xilong Scientific Co. Ltd. (Shantou, China). Mitochondrial Complex I test kit, Mitochondrial Complex II test kit, Mitochondrial Complex III test kit and Mitochondrial Complex IV test kit were supplied by Mlbio Co. Ltd. (Chengdu, China).

### 2.2 Synthesis of derivatives

**FIGURE 1:**
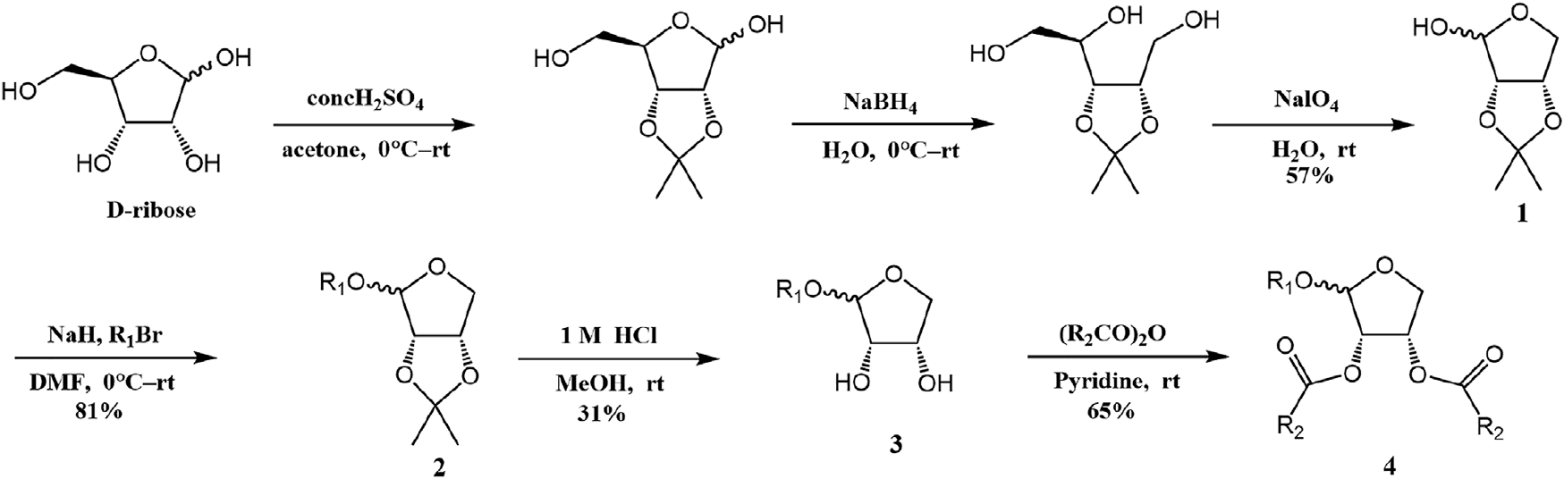
Synthesis of the derivatives of octadecanoic acid-3,4-tetrahydrofuran diester.

The synthesis process is shown in Figure 1. The D-ribose was suspended in acetone under ice-bath, and concentrated sulfuric acid was added for catalysis. The reaction formed ketal at room temperature to protect the 3 and 4 hydroxyl groups. Then, sodium borohydride and water were added under ice-bath. After oxidization by sodium periodate, tetrahydrofuran-3,4-acetone-2-ol (Compound 1) was obtained with yield rate of 57%. Then, the compound 1 was dissolved in dimethyl formamide and followed by addition of sodium hydride and bromide to form compound 2 with yield rate of 89%. Compound 3 (yield rate of 34%) was prepared through aqueous desorption by hydrochloric acid in methanol. Finally, anhydride was added in pyridine and the compound 3 was esterified to obtain the compound 4 (yield rate of 82%).

### 2.3 Collection of mites

*S. scabiei* larvae were collected from toe of infected rabbits (College of Veterinary Science, Sichuan Agricultural University) and then placed in Petri dishes. The dishes were incubated at 35°C for 30 min (Chen et al., 2014). *S. scabiei* larvae that had six legs were easily distinguished from the nymph and adult mites that had eight legs. Then, *S. scabiei* larvae was picked one by one under a stereomicroscope. Motile *S. scabiei* larvae were used in all experiments.

### 2.4 The acaricidal activity

Octadecanoic acid-3,4-tetrahydrofuran diester derivatives were used to perform the acaricidal activity tests against *S. scabiei* larvae. Octadecanoic acid-3,4-tetrahydrofuran diester derivatives were diluted to 20, 10, and 5 mg/ml with liquid paraffin. Polystyrene plates containing 40 mites were soaked in various concentrations of Octadecanoic acid-3,4-tetrahydrofuran diester derivatives. Liquid paraffin was used as the control. Under the same conditions, the plates containing mites were incubated at 25°C with 75% relative humidity and the number of dead mites was computed every minutes for all death.

### 2.5 Transmission electron microscopy observation

Transmission electron microscopy (TEM) assay was performed by using previously established methods (He et al., 2022). Lay larval mites were placed in polystyrene plates, 40 mites per plate. The octadecanoic acid-3,4-tetrahydrofuran diester was derivative diluted with liquid paraffin and added dropwise to the polystyrene plate for pretreatment. Place all plates in a humidity chamber (relative humidity, 75%; temperature, 25°C). After pretreatment for 60 min, mites were washed three times with PBS (pH 7.2) and fixed by pre-cooling glutaraldehyde (2.5%, pH 7.2). The mites were fixed with 1.0% osmic acid and dehydrated with a series concentration of acetone. Ultrathin longitudinal sections were cut, stained with uranyl acetate and lead citrate. The ultrathin sections were observed by a TEM (Abdel-Ghany et al., 2021).

### 2.6 RNA extraction, Illumina sequencing and data analysis

Briefly, the total RNA of mites was extracted with Trizol reagent (Invitrogen) as described by the manufacturer’s instructions, and the concentration and quality of RNA were determined by Agilent 2100 Bioanalyzer (Agilent, Santa Clara, CA, United States). The double stranded cDNA was purified using the qiquick PCR Purification Kit (Qiagen, Germany), and the library was prepared by Illunima HiSeq X-ten for sequencing analysis. The transcriptomic datas were analyzed using the modified protocol previously described (Xu et al., 2021). Cleaned reads of each sample were mapped to the sequenced genome of *S. scabiei* using Bowtie. Reads aligned using Bowtie were assembled into transcripts using Cufflinks, and then merged with Cuffmerge. Differential expression profiles were determined using Cuffdiff (Version: 2.1.1) with default parameters. Hierarchical clustering analysis was performed using Cluster 3.0. The gene expression level is calculated by using RPKM method (Reads Per kb per Million reads).

### 2.7 Determination of enzyme activity

Larval mites were placed in small polystyrene plates. Then, 10 μl octadecanoic acid-3, 4-tetrahydrofuran diester solutions with concentrations of 20 mg/ml, 10 mg/ml, and 5 mg/ml were added, respectively. The solvent control group was also set. All plates were incubated at a humidity chamber (relative humidity, 75%; temperature, 25°C). After 15, 30, and 45 min of treatment, each group of mites were homogenized with 0.25 ml saline in an ice bath. After centrifugation (2500 *g*, 10 min) under 4°C, the supernatant fluid was collected as enzyme extracts. The enzyme activities were then measured according to the manufacturer’s instruction.

### 2.8 Statistical analyses

All results were expressed as mean ± standard deviation. The significance of differences between different concentrations was analyzed by one-way ANOVA, followed by the student’s t test.

## 3 Results

### 3.1 Structure of derivatives

The derivatives of octadecanoic acid-3,4-tetrahydrofuran diester were obtained by substitution at 2-, 3- or 4-position of tetrahydrofuran -3,4-ketal -2-alcohol, and 19 compounds were obtained. The structures of the newly synthesized compounds were deduced from ^1^H and ^13^C NMR spectra (Supplementary Figures S1A–S19B and Supplementary Table S1).

### 3.2 Toxicity evaluation

The octadecanoic acid-3,4-tetrahydrofuran diester and its derivatives were tested for their vitro acaricidal activity, and the results were shown in Table 1. The acaricidal activity of these derivatives is closely related to the types of substituents at the 3 and 4-position in their chemical structures. Specifically, when benzoic acid is used as the substituent at the 3 and 4-position, the median lethal concentration (LC_50_) is the lowest compared with acetic acid, valeric acid, dodecanoic acid, and octadecanoic, which means the acaricidal activity is the strongest. On the other hand, among the different side chain modifications at the 2-position of the furan ring, the derivatives with benzyloxy group at 2-position have the strongest acaricidal activity, while the acaricidal activities of the derivatives substituted with methoxy group, allyloxy group, and pentyloxy group decreased sequentially. The results showed that carbon chain substituents with different lengths at 2-position, 3-position, and 4-position can affect the acaricidal activity of 3,4-tetrahydrofuran diester derivatives. The structure-activity relationship indicated that the shorter the carbon chain length of the substituent, the stronger the acaricidal activity, and the strongest activity was when substituted with a substituent containing a benzene ring. As shown in Table 1, benzoic acid-2-benzyloxy-3,4-tetrahydrofuran diester has strong acaricidal activity, which is twice as much as octadecanoic acid. Basing on the above results, we found that the furan ring substituted with benzyloxy group at the 2-position and formed benzoate at the 3 and 4-position (benzoic acid-2-benzyloxy-3,4-tetrahydrofuran diester) had the strongest acaricidal activity, and it is used as our target drug for further research.

**TABLE 1:** *In vitro* acaricidal activity of 3,4-tetrahydrofuran diester derivatives.

3,4-Tetrahydrofuran diester derivatives	Chemical structural formula	Time for killing the first/min	Time for killing all mites/min	Regression equation	LC_50_ / mg·ml^−1^
Methoxy-2-dodecarbonate-3,4-tetrahydrofuran diester	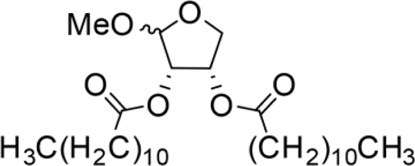	143.33 ± 16.07^A^	750.00 ± 17.32^A^	Y=−2.643 + 2.575X	10.031
Methoxy-2-benzoic acid-3,4-tetrahydrofuran diester	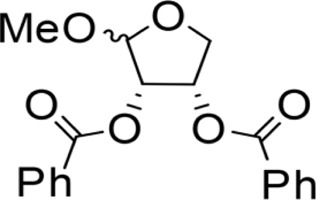	16.66 ± 2.89^B^	134.67 ± 15.28^B^	Y=−2.507 + 1.823X	3.955
Methoxy-2-valeric acid-3,4-tetrahydrofuran diester	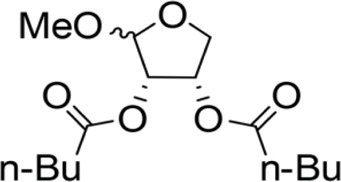	71.67 ± 10.41^C^	643.33 ± 29.63^A^	Y=−2.754 + 2.953X	8.571
Methoxy-2-acetic acid-3,4-tetrahydrofuran diester	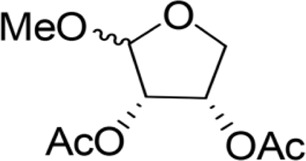	43.33 ± 5.77^C^	233.67 ± 25.17^C^	Y=−1.421 + 1.863X	5.837
Allyloxy-2-octadecarbonate-3,4-tetrahydrofuran diester	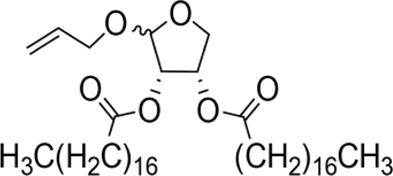	210.33 ± 12.28^A^	920.00 ± 52.43^A^	Y=−3.399 + 2.582X	21.114
Allyloxy-2-dodecarbonate-3,4-tetrahydrofuran diester	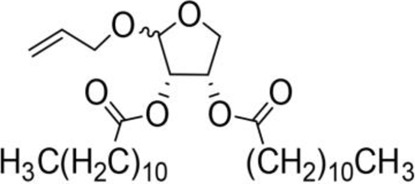	163.33 ± 15.33^A^	823.67 ± 47.68^A^	Y=−3.211 + 2.742x	15.798
Allyloxy-2-benzoic acid-3,4-tetrahydrofuran diester	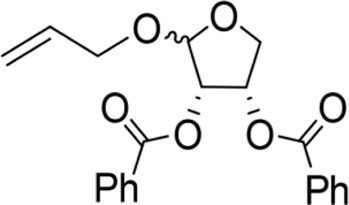	30.66 ± 6.89^B^	214.52 ± 20.07^B^	Y=−3.782 + 1.461X	6.729
Allyloxy-2-valeric acid-3,4-tetrahydrofuran diester	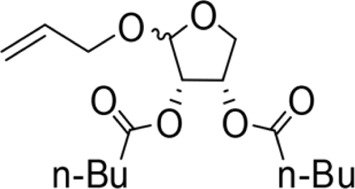	45.33 ± 7.53^B^	315.84 ± 15.28^B^	Y=−3.894 + 3.939X	9.796
Allyloxy-2-acetic acid-3,4-tetrahydrofuran diester	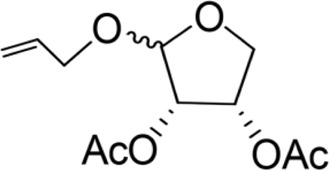	96.67 ± 15.42^C^	725.43 ± 35.38^A^	Y=−2.968 + 2.550X	13.582
Benzyloxy-2-octadecarbonate-3,4-tetrahydrofuran diester	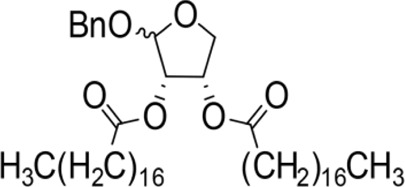	115.33 ± 10.38^A^	520.00 ± 45.83^A^	Y = −7.373 + 6.649X	12.443
Benzyloxy-2-dodecarbonate-3,4-tetrahydrofuran diester	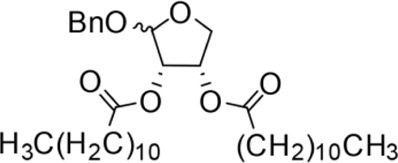	94.33 ± 16.07^A^	450.00 ± 17.32^A^	Y=−8.599 + 8.778X	9.523
Benzyloxy-2-benzoic acid-3,4-tetrahydrofuran diester	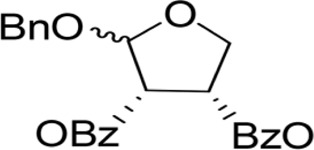	5.66 ± 2.89^B^	45.67 ± 8.28^B^	Y=−0.383 + 2.031X	0.452
Benzyloxy-2- valeric acid-3,4-tetrahydrofuran diester	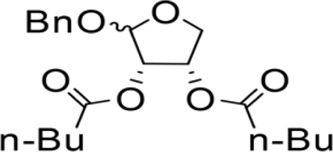	35.67 ± 7.56^C^	420.33 ± 46.63^A^	Y=−1.954 + 2.453X	6.542
Benzyloxy-2-acetic acid-3,4-tetrahydrofuran diester	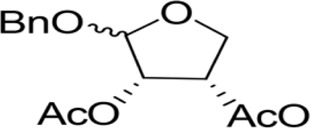	28.33 ± 4.38^C^	210.67 ± 23.17^C^	Y=−2.211 + 1.363X	4.628
Pentoxy-2- octadecarbonate-3,4-tetrahydrofuran diester	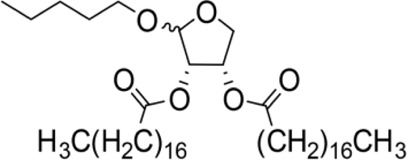	244.33 ± 25.28^A^	1070.00 ± 51.43^A^	Y=−2.017 + 3.782X	25.114
Pentoxy-2- dodecarbonate-3,4-tetrahydrofuran diester	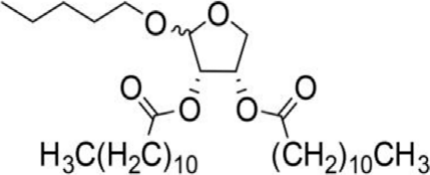	185.33 ± 23.33^A^	870.67 ± 50.68^A^	Y=−2.921 + 2.237X	20.210
Pentoxy-2-benzoic acid-3,4-tetrahydrofuran diester	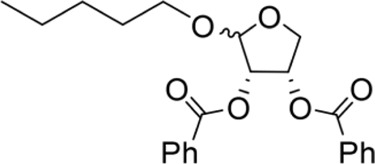	55.66 ± 7.89^B^	287.52 ± 35.07^B^	Y=−3.570 + 3.564X	11.277
Pentoxy-2-valeric acid-3,4-tetrahydrofuran diester	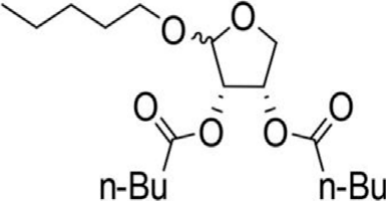	124.67 ± 15.42^C^	795.43 ± 43.38^A^	Y=−3.921 + 3.173X	17.213
Pentoxy-2-acetic acid-3,4-tetrahydrofuran diester	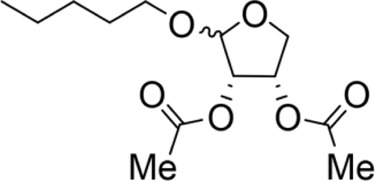	60.33 ± 5.53^B^	420.84 ± 27.28^C^	Y=−4.131 + 3.559X	14.482
Octadecanoic acid-3,3-tetrahydrofuran diester	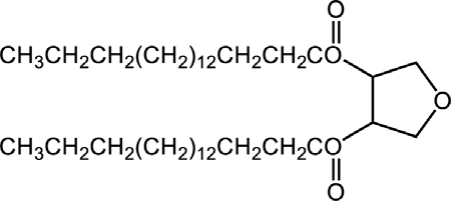	54.00 ± 5.77^A^	1440.00 ± 0.00^E^	—	1.945
Liquid paraffin	—	963.50 ± 15.00^D^	1440.00 ± 0.00^E^	—	—

**FIGURE 2:**
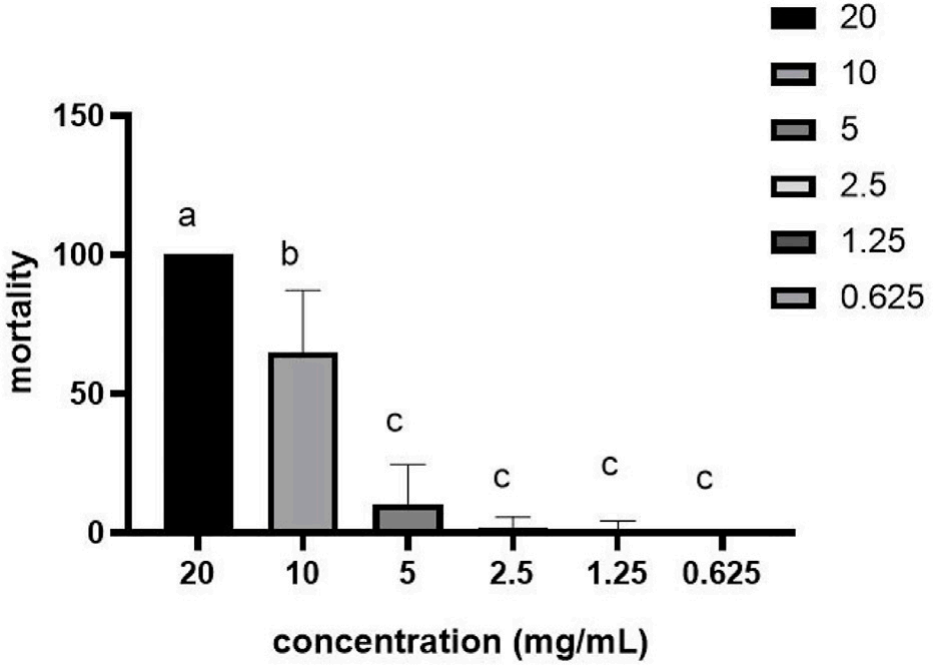
The mortality of benzoic acid-2-benzyloxy-3,4-tetrahydrofuran diester against S. scabiei at 60 min. The difference between data with the different small letters within a column is significant (*p* < 0.05).

The LC_50_ determination results of benzoic acid-2-benzyloxy-3,4-tetrahydrofuran diester on scabies mites are shown in Figure 2. The mortality of scabies mite was significantly correlated with the concentration of the drug (*r* = 0.937, *p* < 0.01). According to the statistical results of the miticidal rate of the drug at 60 min, the obtained virulence regression curve is Y = 1.105X-1.965, which is a linear model. The LC_50_ value of benzoic acid-2-benzyloxy-3,4-tetrahydrofuran diester at 60 min was 5.342 mg/ml, with the 95% confidence limits 4.132–6.775.

The toxicity of benzoic acid-2-benzyloxy-3,4-tetrahydrofuran diester was evaluated using a model of probability regression analysis. The benzoic acid-2-benzyloxy-3,4-tetrahydrofuran diester at three concentrations (20, 10, and 5 mg/ml) was highly toxic to *S. scabiei*. In all tests, significant differences were found between larval mite mortality and the treatment time (*p* < 0.01), which was time-dependent and concentration-dependent. At concentration of 20, 10, and 5 mg/ml, the median lethal time (LT_50_) values of benzoic acid-2-benzyloxy-3,4-tetrahydrofuran diester were 16.138, 47.274, and 108.122 min, respectively and the corresponding correlation coefficients were 0.911, 0.977, and 0.956, respectively (Table 2). The median lethal concentration probit regression equation of the benzoic acid-2-benzyloxy-3,4-tetrahydrofuran diester for *S. scabiei* larvae was Y = 1.105X-1.965. The LC50 value of benzoic acid-2-benzyloxy-3,4-tetrahydrofuran diester to *S. scabiei* larvae was 5.342 mg/ml with 95% confidence limits of 4.132–6.775.

**TABLE 2:** The probit regression analysis of toxicity (LT50) of benzoic acid-2-benzyloxy-3,4-tetrahydrofuran diester against *S. scabiei* var. *cuniculi* larvae *in vitro*.

Concentration (mg/ml)	Regression line	LT50 (95% Fl) (min)	Chi-squared	Correlation coefficient
20	Y=−3.696 + 1.345X	16.138 (13.616–18.606)	18.669	0.911
10	Y=−8.381 + 2.167X	47.274 (40.555–55.563)	28.266	0.977
5	Y=−3.442 + 0.723X	108.122 (82.054–146.919)	30.271	0.956

### 3.3 Transmission electron microscopy observation

**FIGURE 3:**
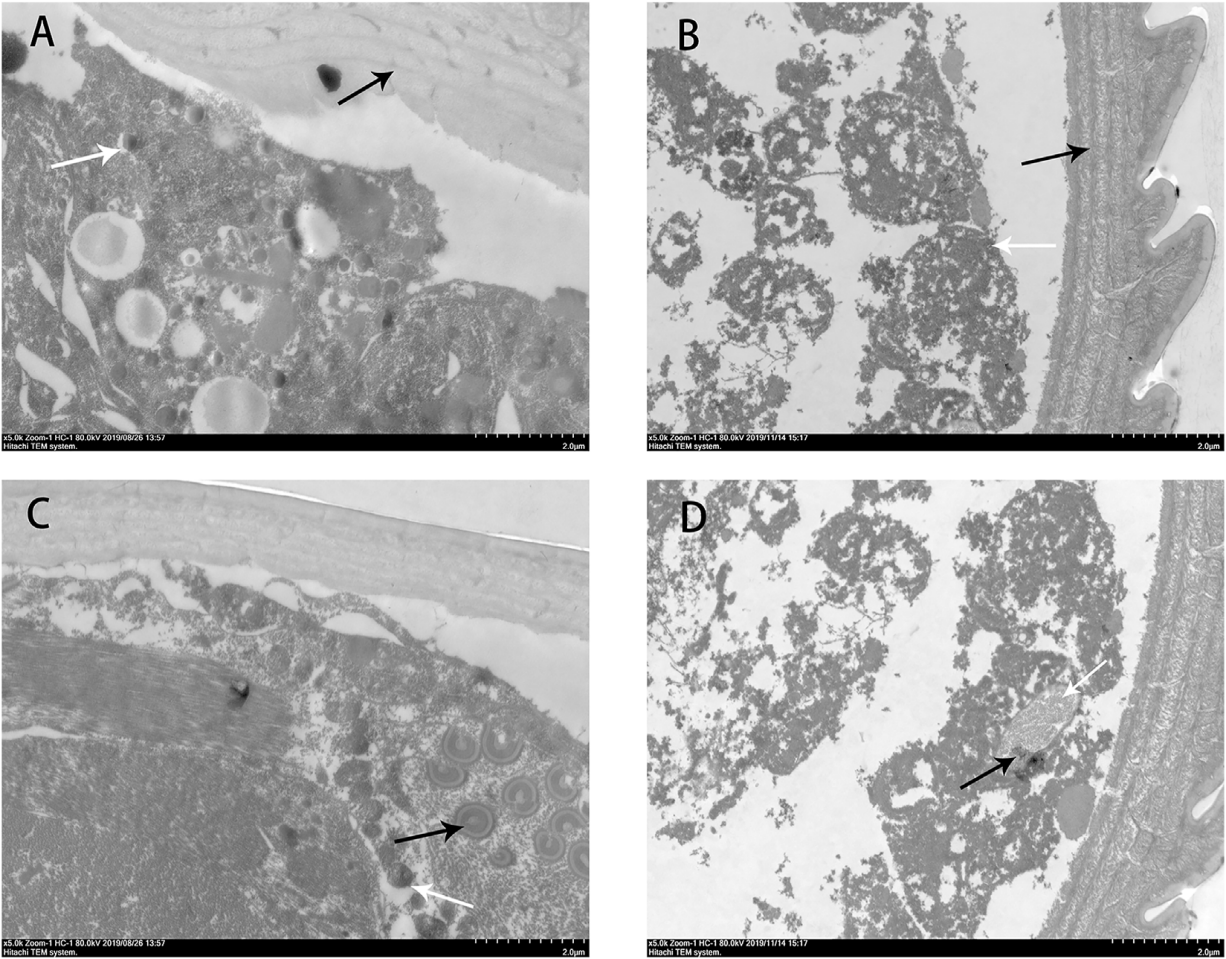
The effect of benzoic acid -2- benzyloxy - 3,4 - tetrahydrofuran diester on S. scabiei mite body wall after 1 h. The control group **(A)** epidermis body walls, the dermatoglyph of waves can see, the nucleus of the corium layer was integrated (×2550). The experimental group **(B)** epidermis body walls, the dermatoglyph of waves becomes smooth, the nucleus meltdown (×2550). The control group **(C)** I-type cells of body cavity, the nucleus was located in cell edge, cytoplasm contained lipid droplets, mitochondria (×6000). The experimental group **(D)** I-type cells of body cavity, the nucleus meltdown, mitochondria damage (×6000).

Under transmission electron microscope, the body wall was visible undulating dermatoglyph, and the skin prickle cell and nucleus of dermis of *S. scabies* mites were complete in the control group (Figure 3A). After treating with benzoic acid-2-benzyloxy-3,4-tetrahydrofuran diester, the dermatoglyph layer became irregular and the lines disappeared, the dermis cell organelles were dissolved, and the nuclear chromatin was ablated (Figure 3B). The control group had the complete I-type body cavity cells structure (Figure 3C). In the experimental group, the cell membrane and nucleus membrane of type -I body cavity were damaged, and the nuclear chromatin were ablated. The dermatoglyph layer was irregular and lines disappeared (Figure 3D).

### 3.4 RNA-seq transcriptomic assay results

Sequencing of mites transcriptome generated 60,872,71 reads. The value of Q30 was above 93.13% in this study, indicating the high availability of the sequencing data. The high quality reads were *de novo* RNA-seq assembly was performed using Trinity 2.5 which produced 34,919 contigs with lengths ≥300 bp.

**FIGURE 4:**
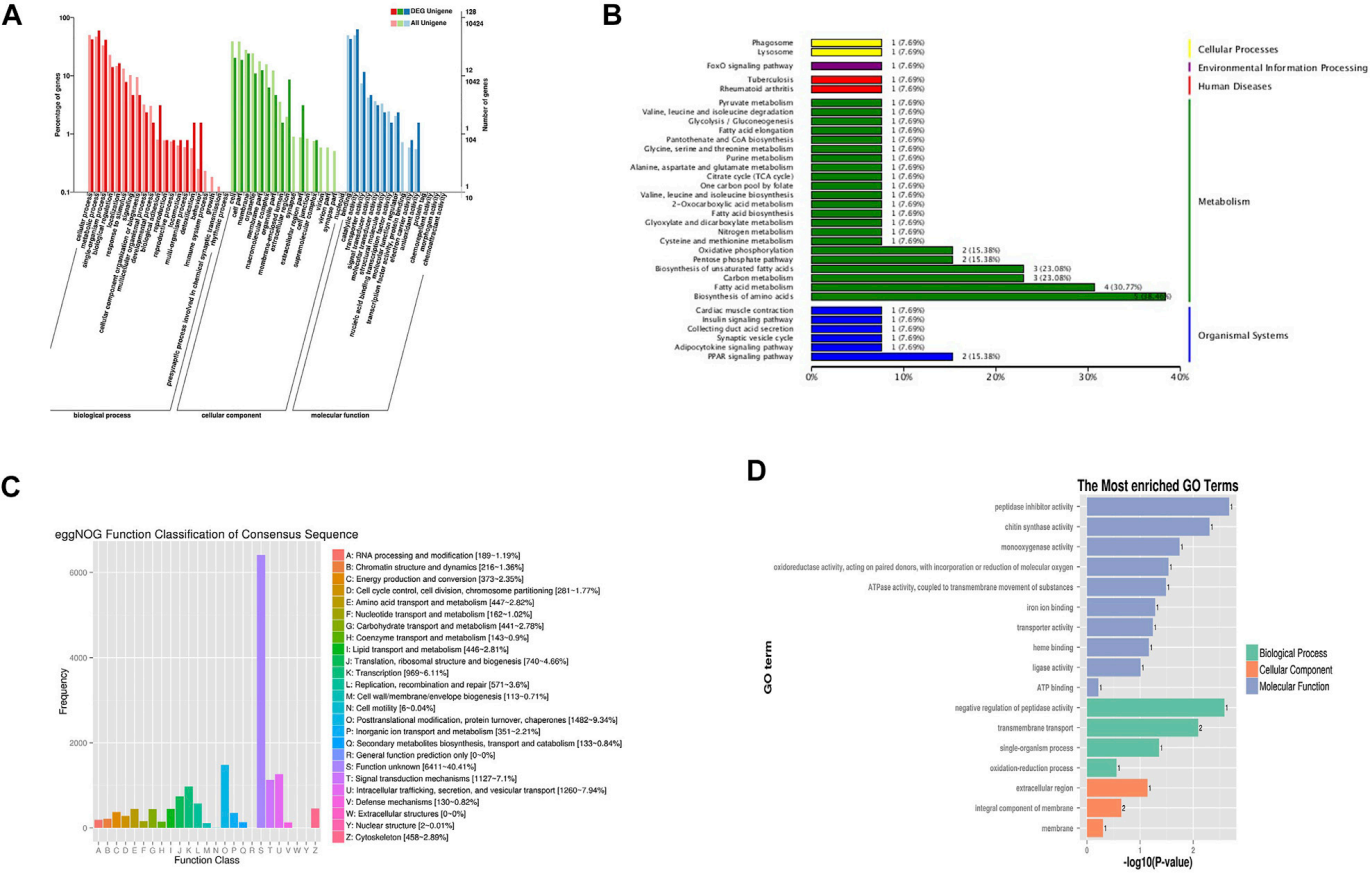
RNA-seq transcriptomic assay results. **(A)** The GO annotation of differentially expressed genes. **(B)** The KEGG pathway annotation of differentially expressed genes. **(C)** The eggNOG function classification of consensus sequence. **(D)** The GO annotation of the most enriched expressed genes.

The differentially expressed genes were identified using DESeq2 (q-value < 0.05). After treatment with benzoic acid-2-benzyloxy-3,4-tetrahydrofuran diester, we found that 281 genes were differentially expressed, of which 211 are down-regulated and 70 are up-regulated. Gene ontologies (GO) terms were assigned to assemble unigenes and describe the related processes of gene products (Biological process, Molecular function and Cellular component) (Figure 4A). GO annotation indicated that the function of differentially expressed genes mainly distributed in several categories, such as “oxidation-reduction process,” “protein catabolic process” and “coenzyme metabolic process.” In addtion, “oxidative phosphorylation process,” “oxidoreductase activity,” “ATPase activity” were prominently represented. (Figure 4D). There were 15,863 Unigenes in the transcriptome of scabies and corresponding relationships with the eggNOG database. According to their functions, Unigenes can be divided into 25 categories (Figure 4C). They mainly included “energy production and conversion,” “Amino acid transport and metabolism” and “Lipid transport and metabolism.” Among the 122 differentially expressed genes were mapped to 244 KEGG pathways within 86 categories, and 31 pathways (six pathways in “Metabolism”) were significantly enriched, including “Pentose phosphate pathway,” “Glycolysis/Gluconeogenesis,” “Oxidative phosphorylation,” “Fatty acid biosynthesis,” and “Purine metabolism” (Figure 4B). Enrichment analysis results indicated that benzoic acid-2-benzyloxy-3,4 -tetrahydrofuran diester could affect the growth of mites by regulating the expression of related metabolic genes.

### 3.5 Determination of mitochondrial complex activity

#### 3.5.1 The mitochondrial complex I activity

**FIGURE 5:**
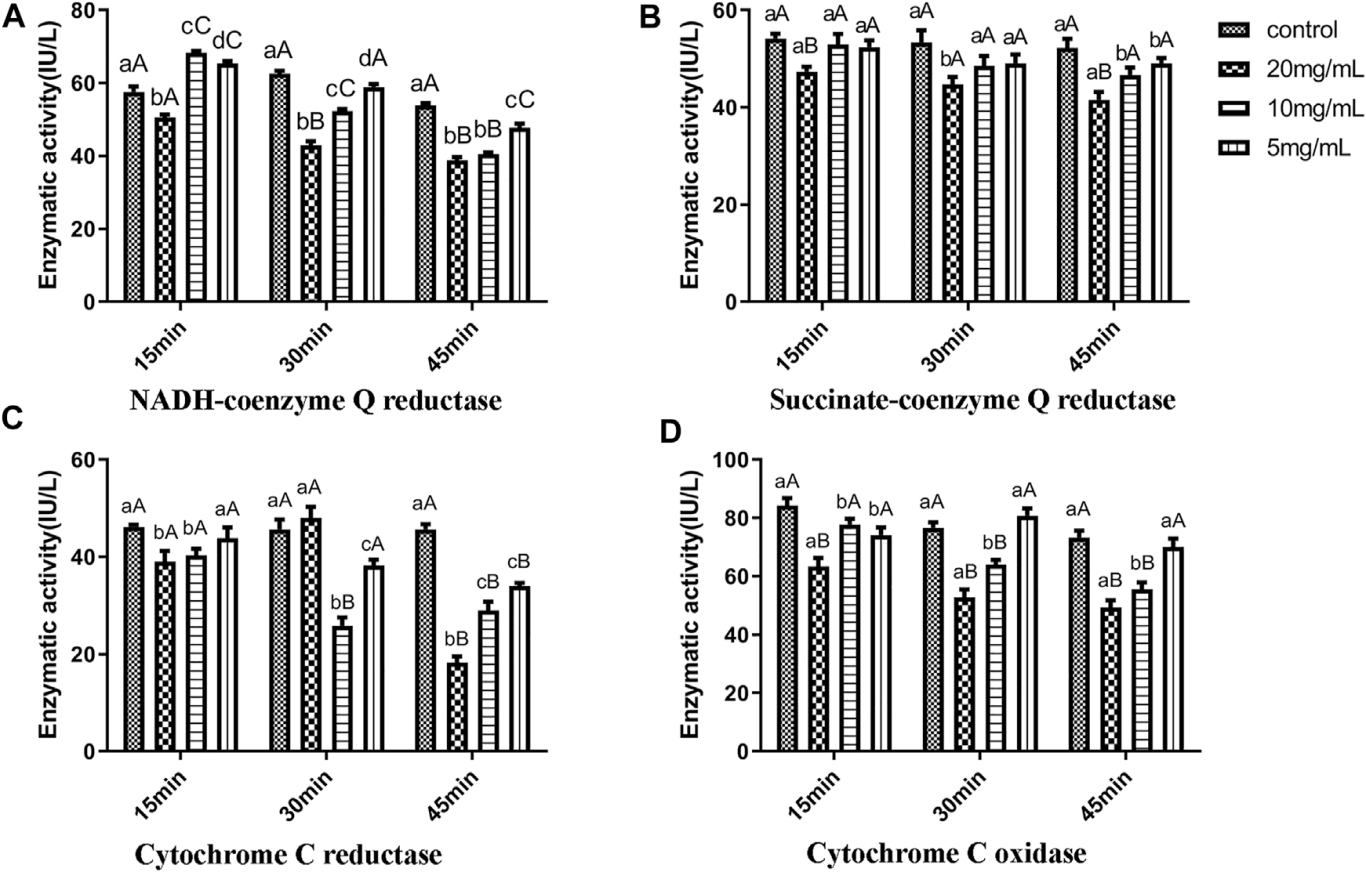
The changes of enzyme activity of scabies mites treated for 15, 30, and 45 min. **(A)** Mitochondrial Complex I activity; **(B)** Mitochondrial Complex II activity; **(C)** Mitochondrial Complex III activity; **(D)** Mitochondrial Complex IV activity. The difference between data with the different small letters within a column is significant (*p* < 0.05), and the difference between data with the different capital letters is at the (*p* < 0.01) level.

The changes of the Mitochondrial Complex I (NADH-coenzyme Q reductase) activity in larval mites treated with different concentration of benzoic acid-2-benzyloxy-3,4-tetrahydrofuran diester were shown in Figure 5A. The Mitochondrial Complex I activity of larval mites treated with 20 mg/ml benzoic acid-2-benzyloxy-3,4-tetrahydrofuran diester was lower than that of the control group throughout 15–45 min (*p* < 0.05). However, the activity of larvae treated with benzoic acid-2-benzyloxy-3,4-tetrahydrofuran diester at 10 and 5 mg/ml showed a trend of increasing first and then decreasing. The inhibition rate of Mitochondrial Complex I activity was ranged from 14.02% to 38.65% in comparison with the control group at a concentration of 20 mg/ml. The activity of Mitochondrial Complex I increased by 18.58% after 10 mg/ml benzoic acid-2-benzyloxy-3,4-tetrahydrofuran diester was treated for 15 min, while it was inhibited by 32.70% at 45 min. The same trend was observed in the activity of larvae treated with 10 and 5 mg/ml benzoic acid-2-benzyloxy-3,4-tetrahydrofuran diester. The activity of larvae treated with 5 mg/ml benzoic acid-2-benzyloxy-3,4-tetrahydrofuran diester increased by 13.50% at 15 min and decreased by 12.64% at 45 min. The results showed that 20 mg/ml benzoic acid-2-benzyloxy-3,4-tetrahydrofuran diester would inhibit the Mitochondrial Complex I activity of larval mites.

#### 3.5.2 The mitochondrial complex II activity

The Mitochondrial Complex II (Succinate-coenzyme Q reductase) activity of larval mites treated with benzoic acid-2-benzyloxy-3,4-tetrahydrofuran diester are shown in Figure 5B. The Mitochondrial Complex II activity of larvae treated with 20, 10, and 5 mg/ml benzoic acid-2-benzyloxy-3,4-tetrahydrofuran diester was lower than that of the control group at 15–45 min during the whole experiment. The inhibition rates of Mitochondrial Complex II activity were ranged from 2.3% to 12.56% with treatment for 15 min, from 7.04% to 15.43% with treatment for 30 min, from 12.04% to 23.58% with treatment for 45 min. The results implicated that the Mitochondrial Complex II activity of larval mites related to the treatment time with benzoic acid-2-benzyloxy-3,4-tetrahydrofuran diester.

#### 3.5.3 The mitochondrial complex III activity

The Mitochondrial Complex III (Cytochrome C reductase) activity in larval mites treated with benzoic acid-2-benzyloxy-3,4-tetrahydrofuran diester are shown in Figure 5C. The activity of Mitochondrial Complex III in larvae treated with 10 and 5 mg/ml benzoate-2-benzyloxy-3,4-tetrahydrofuran diester was lower than that in the control group at each test point during the experiment (*p* < 0.05). The inhibition rates of Mitochondrial Complex III activity were ranged from 5.08% to 12.86% with treatment for 15 min, from 16.45% to 43.50% with treatment for 30 min, from 25.67% to 36.48% with treatment for 45 min. While the Mitochondrial Complex III activity of larval mites treated with 20 mg/ml benzoic acid-2-benzyloxy-3,4-tetrahydrofuran diester was inhibited 15.51% and 66.55% at 15 and 45 min respectively. The results showed that when treated with 20 mg/ml benzoate-2-benzyloxy-3,4-tetrahydrofuran diester, the Mitochondrial Complex III activity was increased firstly and then decreased. However, 10 and 5 mg/ml benzoic acid-2-benzyloxy-3,4-tetrahydrofuran diester would inhibit the Mitochondrial Complex III activity of larval mites.

#### 3.5.4 The mitochondrial complex IV activity

The Mitochondrial Complex IV (Cytochrome C oxidase) activity in larval mites treated with benzoic acid-2-benzyloxy-3,4-tetrahydrofuran diester are shown in Figure 5D. Compared with the control group, the Mitochondrial Complex IV activity of larvae at all test points decreased during the experiment. The decreased rates of Mitochondrial Complex IV activity were ranged from 7.57% to 24.77% with treatment for 15 min, from 5.71% to 31.08% with treatment for 30 min, from 4.46% to 32.79% with treatment for 45 min, respectively. The results showed that the Mitochondrial Complex IV activity of larval mites related to the treatment time of benzoic acid-2-benzyloxy-3,4-tetrahydrofuran diester.

## 4 Discussion

In this study, a series of derivatives were obtained by structural modification of octadecanoic acid-3,4-tetrahydrofuran diester, and their acaricidal activities were tested. It was found that the introduction of benzyloxy substitution at the 2-position of the furan ring and the formation of benzoate at the 3 and 4-position of the furan ring could enhance the acaricidal activity. Benzoic acid-2-benzyloxy-3,4-tetrahydrofuran diester killed the mites quickly and efficiently compared with octadecanoic acid-3,4-tetrahydrofuran diester. The mites using 20 mg/ml benzoic acid-2-benzyloxy-3,4-tetrahydrofuran diester started dying at 5 min and caused 100% mortality in the test mites at 45 min. However, pyrethrin and ivermectin yielded 100% mortality for 825 and 125 min, respectively. The acaricidal activity of 20 mg/ml benzoic acid-2-benzyloxy-3,4-tetrahydrofuran diester had significant effects than ivermectin (20 mg/ml, *p* < 0.01) against *S. scabiei* larvae. The results indicated that benzoic acid-2-benzyloxy-3,4- tetrahydrofuran diester had a strong acaricidal activity against *S. scabiei*.

Benzoic acid-2-benzyloxy-3,4-tetrahydrofuran diester was obtained by structural modification of octadecanoic acid-3,4-tetrahydrofuran diester. Although it has good acaricidal activity, the insecticidal mechanism of benzoic acid-2-benzyloxy-3,4-tetrahydrofuran diester is still unknown. Therefore, in this study, we identified the target of the compound by electron microscopy, RNA-seq transcriptomics and mitochondrial complex enzyme activity changes.

Mitochondrial damages will cause the disorder of rely on ATP energy metabolism, which affects the aerobic respiration of insects, resulting in the deficiency of organism functions and death. Benzoic acid-2-benzyloxy-3,4-tetrahydrofuran diester affected the activities of ATPase, thereby causing the body damage in the mites. Mitochondria is an important and unique organelle in eukaryotic cells and an important site for oxidative phosphorylation and ATP formation. Its function is to provide the “power” for the cell to carry on the energy conversion, supply the energy that the cell needs to carry out various life activities and participate in the synthesis of fatty acids and some proteins (Christophe et al., 2019; Vercellino and Sazanov, 2022). Mitochondria is the storage and supply of cellular energy. Under normal physiological conditions, more than 80% of energy in the body is supplied by mitochondrial oxidative phosphorylation (Kadenbach, 2020). Therefore, mitochondria may become the target of acaricidal drugs, affecting mitochondrial function and ultimately leading to the death of mites. In this study, the results of RNA-seq transcriptomics showed that there were five differentially expressed down-regulated genes in the fatty acid metabolismpaths and four differentially expressed down-regulated genes in oxidative phosphorylation pathway after treatment. After treatment, the down-regulated gene expressions of the biosynthesis of amino acids paths and oxidative phosphorylation pathway subunit may reduce the breakdown of fatty acids and inhibit the production of ATP.

NADH-dehydrogenase (Complex I) is the first enzyme known as electron carriers in the respiratory chain, which can be oxidized by Coenzyme Q10 (Parey et al., 2020). Succinate dehydrogenase (Complex II), has been considered a with the unique property to participate in electron transport chain (Moosavi et al., 2019). Ubiquinol-cytochrome C reductase (Complex III), can catalyze coenzyme Q oxidation and reduction process of cytochrome C. Besides, the enzyme plays an indispensable role in donating electron from QH2 to cytochrome C receptor (Tormos et al., 2011; Lothar et al., 2016). Cytochrome C oxidase, also called “Complex IV,” is the last protein known as electron carriers in the transport chain (Lothar et al., 2016). The energy released during the process of electron transfer, driving the complex I, II, III and IV will proton across a membrane to the clearance of mitochondrial membrane, the proton gradient and electric potential to drive the generation of ATP. The finding in this paper indicated that the decline of the complex I, II, III and IV activity of larval mites treated with benzoic acid-2-benzyloxy-3,4-tetrahydrofuran diester caused the transmembrane transport of protons and electrons in mitochondria affects the production of ATP and then made larval mites starved of energy and dead. Meanwhile, the decline of the complex I, II, III and IV activity also led to the destruction of mitochondria. The finding consists with the observations in RNA-seq transcriptomics and transmission electron microscopy observation.

## 5 Conclusion

Benzoic acid-2-benzyloxy-3,4-tetrahydrofuran diester is an efficient compound against *S. scabiei* var. *cuniculi*. Its acaricidal mechanism is mainly attributed to the interference of energy metabolism, including pentose phosphate pathway, glycolysis/gluconeogenesis, oxidative phosphorylation, and other energy metabolism processes. Benzoic ac-id-2-benzyloxy-3,4-tetrahydrofuran diester can affect ATP production by altering the activity of mitochondrial complexes I, II, III and IV, leading to energy metabolism disorders and death.

## Data Availability

The original contributions presented in the study are included in the article/Supplementary Material, further inquiries can be directed to the corresponding author.

## References

[B1] Abdel-Ghany H. S. M. Abdel-Shafy S. Abuowarda M. El-Khateeb R. M. Hoballah E. M. Fahmy M. M. (2021). Acaricidal Activity of Artemisia Herba-Alba and Melia Azedarach Oil Nanoemulsion against Hyalomma Dromedarii and Their Toxicity on Swiss Albino Mice. Exp. Appl. Acarol. 84, 241–262. 10.1007/s10493-021-00618-2 33934282

[B2] Chen Z. Z. Deng Y. X. Yin Z. Q. Wei Q. Li M. Jia R. Y. (2014). Studies on the Acaricidal Mechanism of the Active Components from Neem (Azadirachta indica) Oil against Sarcoptes Scabiei Var. cuniculi. Vet. Parasitol. 204, 323–329. 10.1016/j.vetpar.2014.05.040 24974121

[B3] Christophe W. Ulrich B. Carola H. Volker Z. (2019). Structure and Function of Mitochondrial Complex I. Biochim Biophys Acta. 1857 (7), 902–914. 10.1016/j.bbabio.2016.02.013 26921811

[B4] Deng Y. Shi D. Yin Z. Guo J. Jia R. Xu J. (2012). Acaricidal Activity of Petroleum Ether Extract of Neem ( Azadirachta indica ) Oil and its Four Fractions Separated by Column Chromatography against Sarcoptes Scabiei Var. cuniculi Larvae In Vitro. Exp. Parasitol. 130, 475–477. 10.1016/j.exppara.2012.02.007, 22349080

[B5] D’Ovidio D. Santoro D. (2021). Efficacy of Fluralaner in the Treatment of Sarcoptic Mange (Sarcoptes Scabiei) in 12 Pet Rabbits. Top. Companion Anim. Med. 43, 100528. 10.1016/j.tcam.2021.100528 33548547

[B6] Du Y. H. Jia R. Y. Yin Z. Q. Pu Z. H. Lu Y. Yang F. (2008). Acaricidal Activity of Extracts of Neem (Azadirachta indica) Oil against the Larvae of the Rabbit Mite Sarcoptes Scabiei Var. cuniculi In Vitro. Vet. Parasitol. 157, 144–148. 10.1016/j.vetpar.2008.07.011 18752898

[B7] Du Y. H. Li J. L. Jia R. Y. Yin Z. Q. Li X. T. Lv C. (2009). Acaricidal Activity of Four Fractions and Octadecanoic Acid-Tetrahydrofuran-3, 4-diyl Ester Isolated from Chloroform Extracts of Neem (Azadirachta indica) Oil against Sarcoptes Scabiei Var. cuniculi Larvae In Vitro. Vet. Parasitol. 163, 175–178. 10.1016/j.vetpar.2009.04.002 19443124

[B8] He Y. Du G. Xie S. Long X. Sun G. Zhu S. (2022). The Insecticidal Efficacy and Physiological Action Mechanism of a Novel Agent GC16 against Tetranychus Pueraricola (Acari: Tetranychidae). Insects 13, 433. 10.3390/insects13050433 35621769 PMC9146473

[B9] Kadenbach B. (2020). Complex IV – the Regulatory Center of Mitochondrial Oxidative Phosphorylation. Mitochondrion 58, 296–302. 10.1016/j.mito.2020.10.004 33069909

[B10] Lothar E. Fei Z. Yihui Y. Xiao Y. Tang W. K. Yu C. A. (2016). Hydrogen Bonding to the Substrate Is Not Required for Rieske Iron-Sulfur Protein Docking to the Quinol Oxidation Site of Complex III. J. Biol. Chem. 291, 25019–25031. 10.1074/jbc.M116.744391 27758861 PMC5122771

[B11] Moosavi B. Zhu X. L. Yang W. C. Yang G. F. (2019). Genetic, Epigenetic and Biochemical Regulation of Succinate Dehydrogenase Function. Biol. Chem. 401, 319–330. 10.1515/hsz-2019-0264 31408429

[B12] Parey K. Wirth C. Vonck J. Zickermann V. (2020). Respiratory Complex I — Structure, Mechanism and Evolution. Curr. Opin. Struct. Biol. 63, 1–9. 10.1016/j.sbi.2020.01.004 32058886

[B13] Seddiek S. A. Khater H. F. El-Shorbagy M. M. Ali A. M. (2013). The Acaricidal Efficacy of Aqueous Neem Extract and Ivermectin against Sarcoptes Scabiei Var. cuniculi in Experimentally Infested Rabbits. Parasitol. Res. 112, 2319–2330. 10.1007/s00436-013-3395-2 23572045

[B14] Song X. Chen Z. Jia R. Cao M. Zou Y. Li L. (2017). Transcriptomics and Proteomic Studies Reveal Acaricidal Mechanism of Octadecanoic Acid-3, 4 - Tetrahydrofuran Diester against Sarcoptes Scabiei Var. cuniculi. Sci. Rep. 7, 45479. 10.1038/srep45479 28361965 PMC5374447

[B15] Tormos K. Anso E. Hamanaka R. Eisenbart J. Joseph J. Kalyanaraman B. (2011). Mitochondrial Complex III ROS Regulate Adipocyte Differentiation. Cell. Metab. 14, 537–544. 10.1016/j.cmet.2011.08.007 21982713 PMC3190168

[B16] Vercellino I. Sazanov L. A. (2022). The Assembly, Regulation and Function of the Mitochondrial Respiratory Chain. Nat. Rev. Mol. Cell. Biol. 23, 141–161. 10.1038/s41580-021-00415-0 34621061

[B17] Xu Z. Qi C. Zhang M. Liu P. Zhang P. He L. (2021). Transcription Response of Tetranychus Cinnabarinus to Plant-Mediated Short-Term and Long -term Selenium Treatment. Chemosphere 263, 128007. 10.1016/j.chemosphere.2020.128007 33297040

