# Supplementary material for: Structural modification of octadecanoic acid-3,4-tetrahydrofuran diester and the acaricidal activity and mechanism of its derivatives against Sarcoptes scabiei var. Cuniculi
Source: Front Pharmacol. 2022 Aug 22;13:953284. doi: 10.3389/fphar.2022.953284 (PMC9442034; doi:10.3389/fphar.2022.953284)
Supplement: Supplementary file 1 [file Table1.docx]

**Supplementary Table 1** The yield of 3,4-tetrahydrofuran diester derivatives

| 3,4-tetrahydrofuran diester derivatives | Chemical structural formula | Yield |
| --- | --- | --- |
| Methoxy-2-dodecarbonate-3,4-tetrahydrofuran diester | 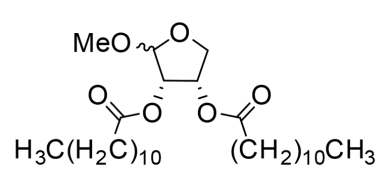 | 69% |
| Methoxy-2-benzoic acid-3,4-tetrahydrofuran diester | 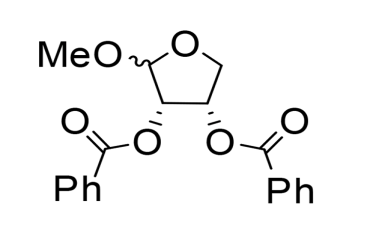 | 73% |
| Methoxy-2-valeric acid-3,4-tetrahydrofuran diester | 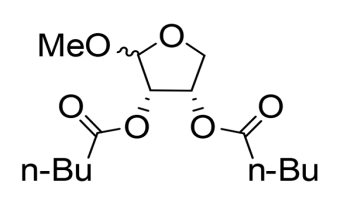 | 69% |
| Methoxy-2-acetic acid-3,4-tetrahydrofuran diester | 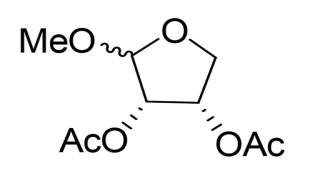 | 78% |
| Allyloxy-2-octadecarbonate-3,4-tetrahydrofuran diester | 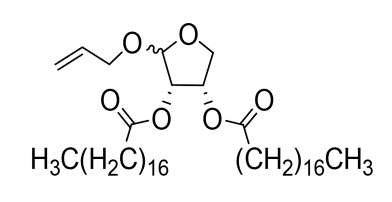 | 66% |
| Allyloxy-2-dodecarbonate-3,4-tetrahydrofuran diester | 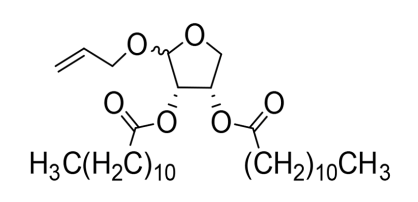 | 72% |
| Allyloxy-2-benzoic acid-3,4-tetrahydrofuran diester | 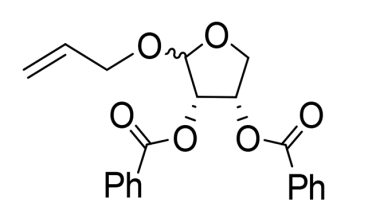 | 78% |
| Allyloxy-2-valeric acid-3,4-tetrahydrofuran diester | 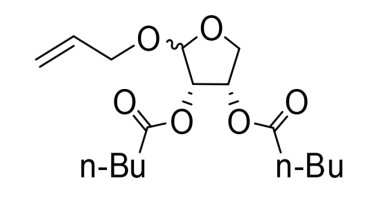 | 81% |
| Allyloxy-2-acetic acid-3,4-tetrahydrofuran diester | 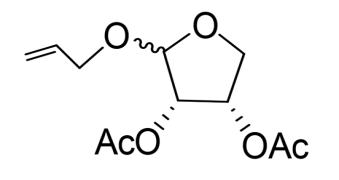 | 81% |
| Benzyloxy-2-octadecarbonate-3,4-tetrahydrofuran diester | 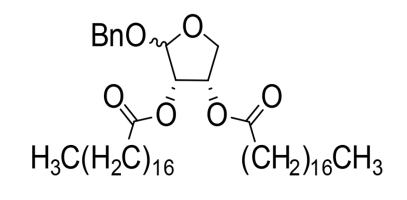 | 71% |
| Benzyloxy-2-dodecarbonate-3,4-tetrahydrofuran diester | 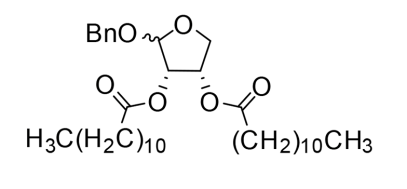 | 71% |
| Benzyloxy-2-benzoic acid-3,4-tetrahydrofuran diester | 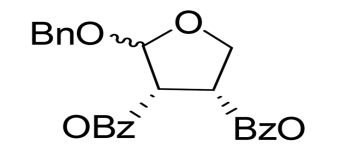 | 71% |
| Benzyloxy-2- valeric acid-3,4-tetrahydrofuran diester | 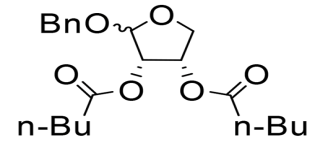 | 74% |
| Benzyloxy-2-acetic acid-3,4-tetrahydrofuran diester | 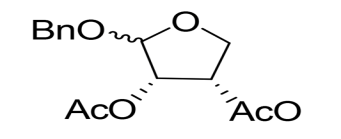 | 72% |
| Pentoxy-2- octadecarbonate-3,4-tetrahydrofuran diester | 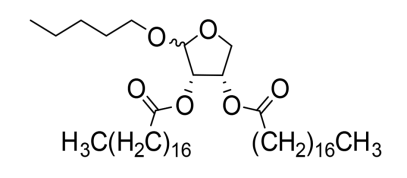 | 71% |
| 71%Pentoxy-2- dodecarbonate-3,4-tetrahydrofuran diester | 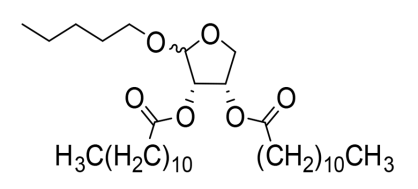 | 741% |
| Pentoxy-2-benzoic acid-3,4-tetrahydrofuran diester | 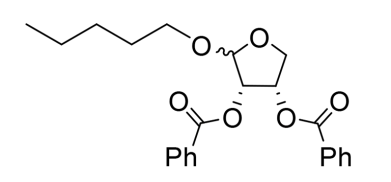 | 71% |
| Pentoxy-2-valeric acid-3,4-tetrahydrofuran diester | 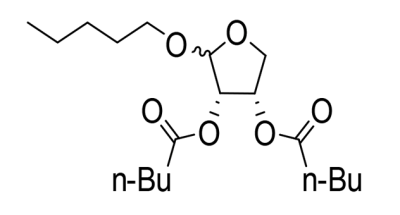 | 74% |
| Pentoxy-2-acetic acid-3,4-tetrahydrofuran diester | 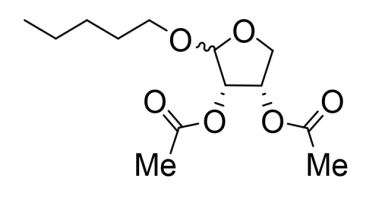 | 72% |
