# Supplementary figures and images for: Structural modification of octadecanoic acid-3,4-tetrahydrofuran diester and the acaricidal activity and mechanism of its derivatives against Sarcoptes scabiei var. Cuniculi
Source: Front Pharmacol. 2022 Aug 22;13:953284. doi: 10.3389/fphar.2022.953284 (PMC9442034; doi:10.3389/fphar.2022.953284)

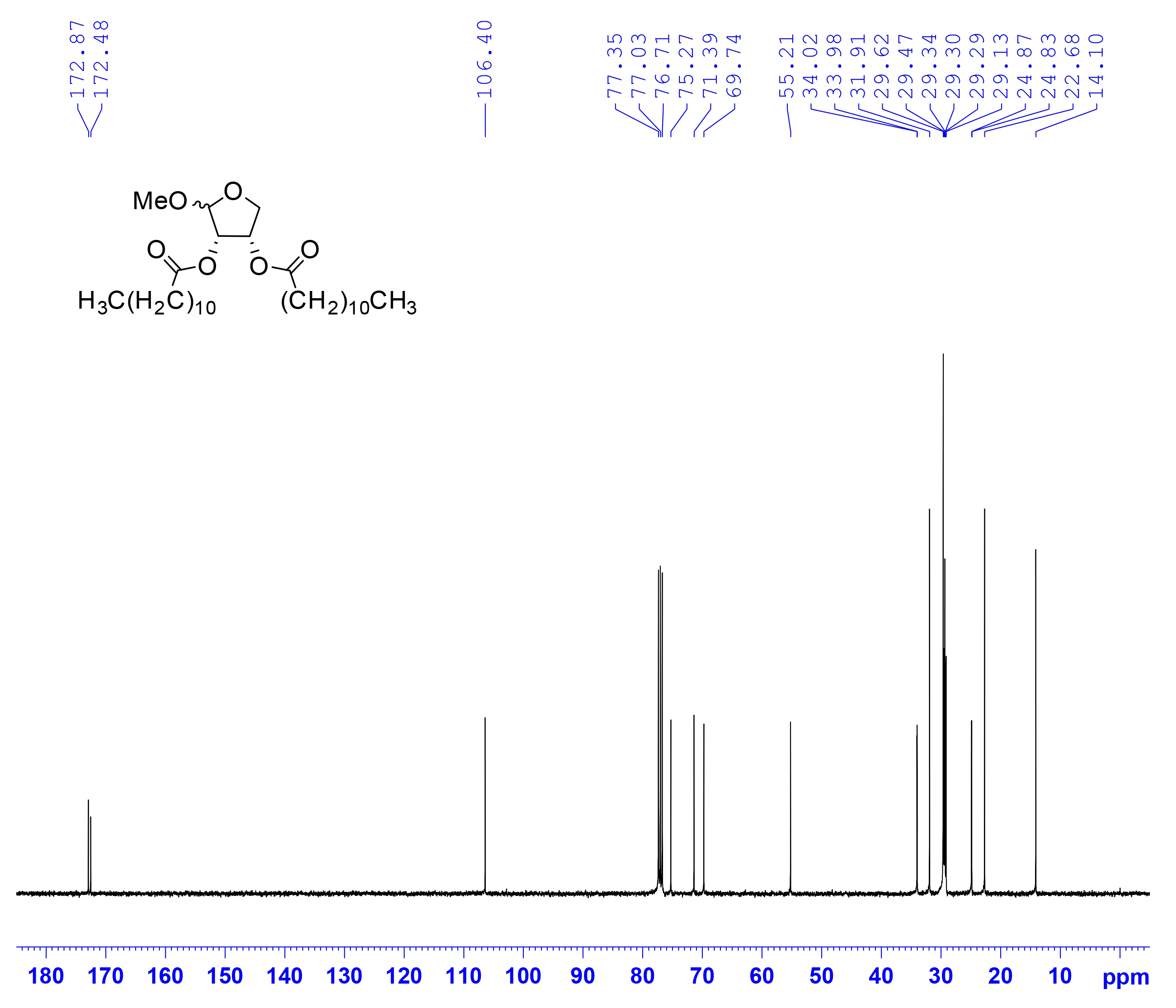

Supplement: Supplementary file 2 [file DataSheet1.ZIP › Supplementary Figure 1. 13C-NMR Methoxy-2-dodecarbonate-3,4-tetrahydrofuran diester.tif]

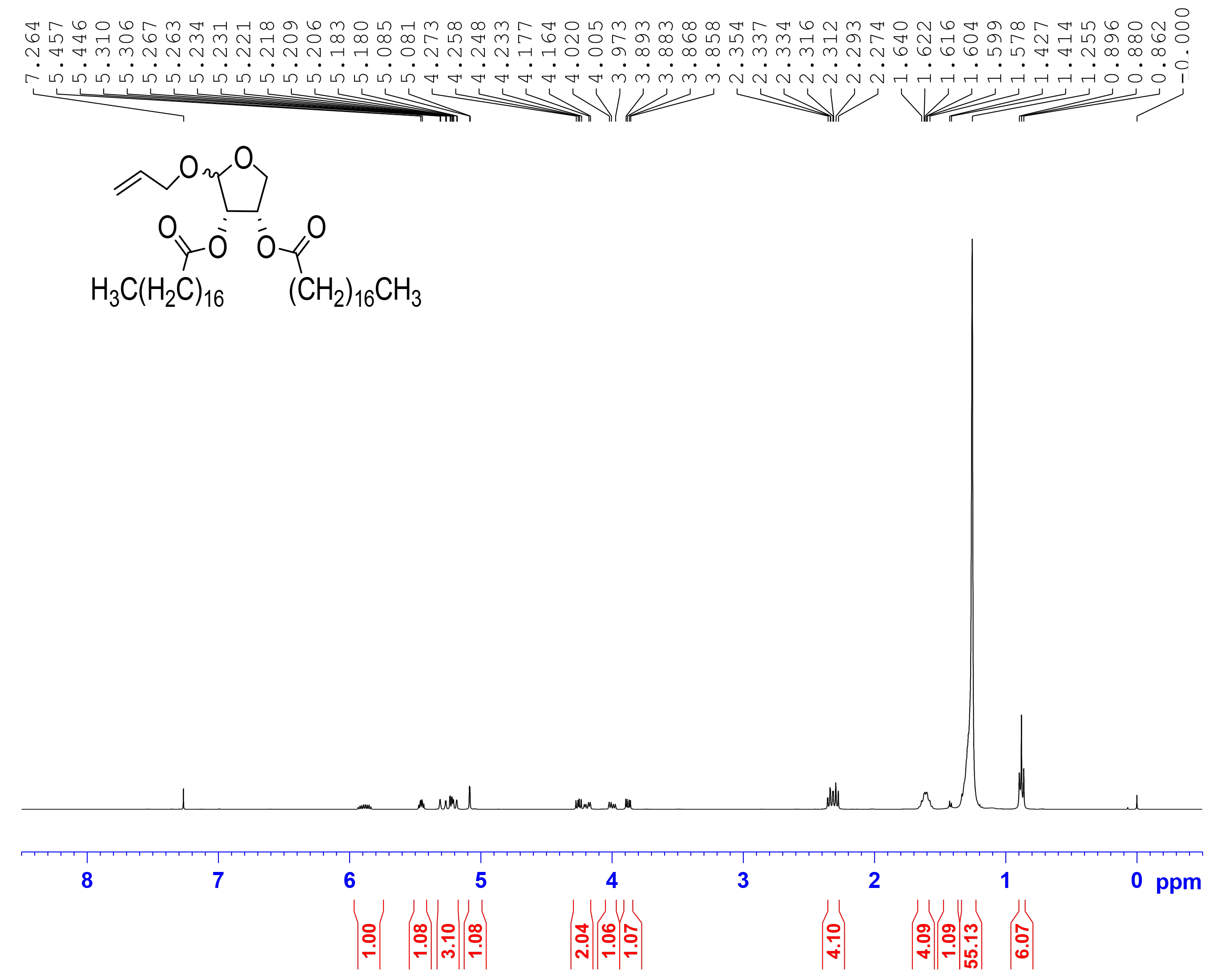

Supplement: Supplementary file 2 [file DataSheet1.ZIP › Supplementary Figure 10. 1H-NMR Allyloxy-2-octadecarbonate-3,4-tetrahydrofuran diester.tif]

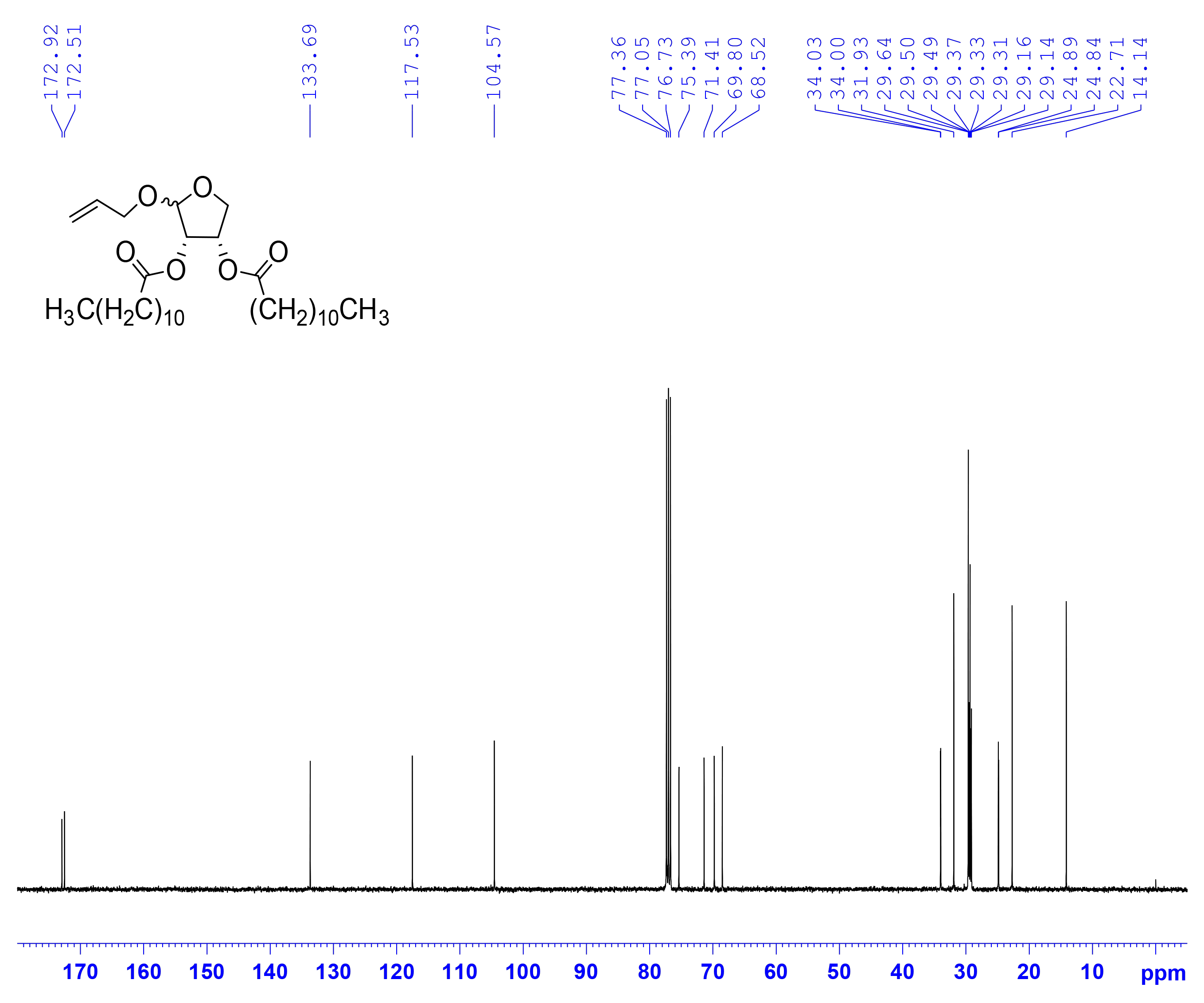

Supplement: Supplementary file 2 [file DataSheet1.ZIP › Supplementary Figure 11. 13C-NMR Allyloxy-2-dodecarbonate-3,4-tetrahydrofuran diester.tif]

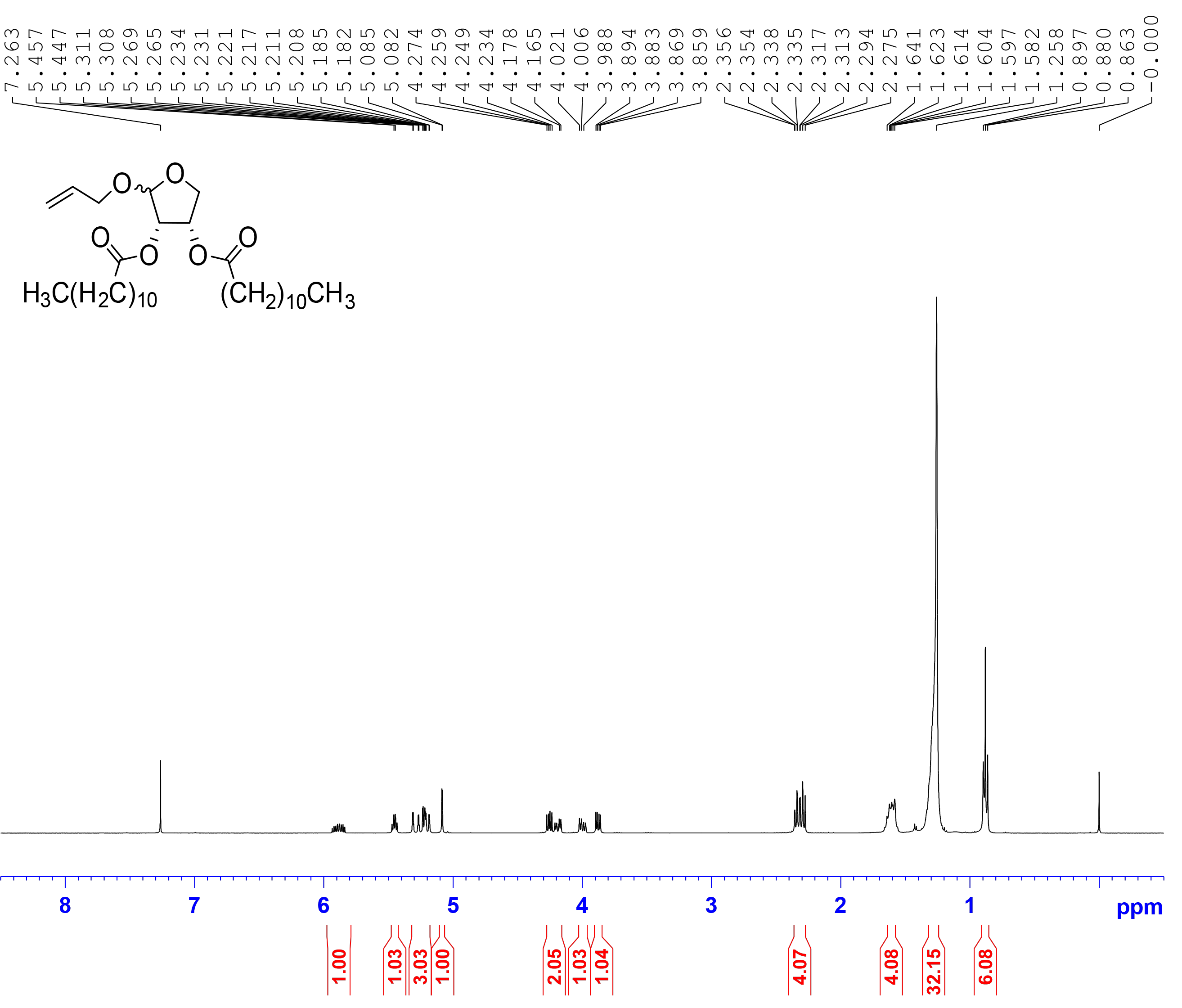

Supplement: Supplementary file 2 [file DataSheet1.ZIP › Supplementary Figure 12. 1H-NMR Allyloxy-2-dodecarbonate-3,4-tetrahydrofuran diester.tif]

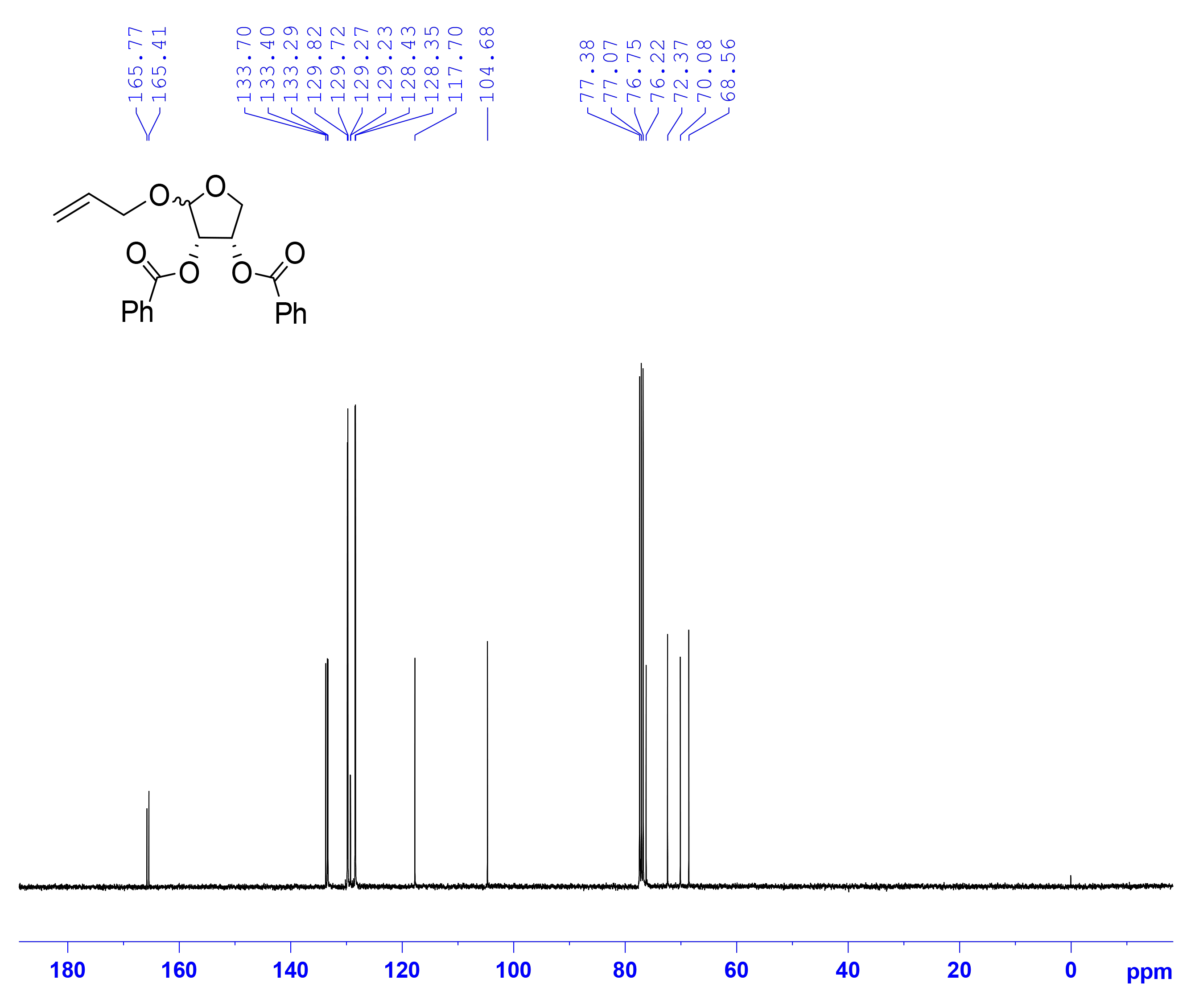

Supplement: Supplementary file 2 [file DataSheet1.ZIP › Supplementary Figure 13. 13C-NMR Allyloxy-2-benzoic acid-3,4-tetrahydrofuran diester.tif]

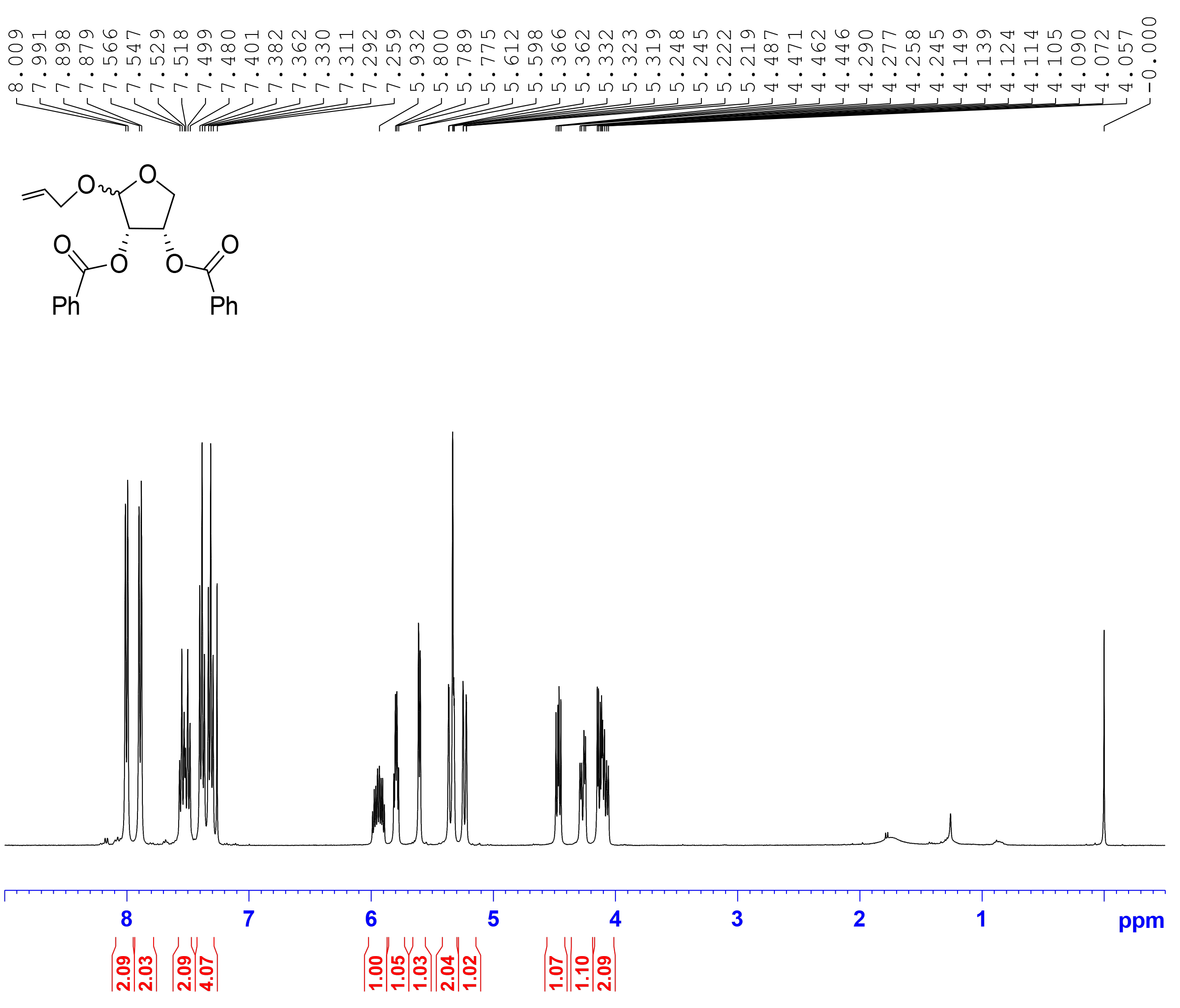

Supplement: Supplementary file 2 [file DataSheet1.ZIP › Supplementary Figure 14. 1H-NMR Allyloxy-2-benzoic acid-3,4-tetrahydrofuran diester.tif]

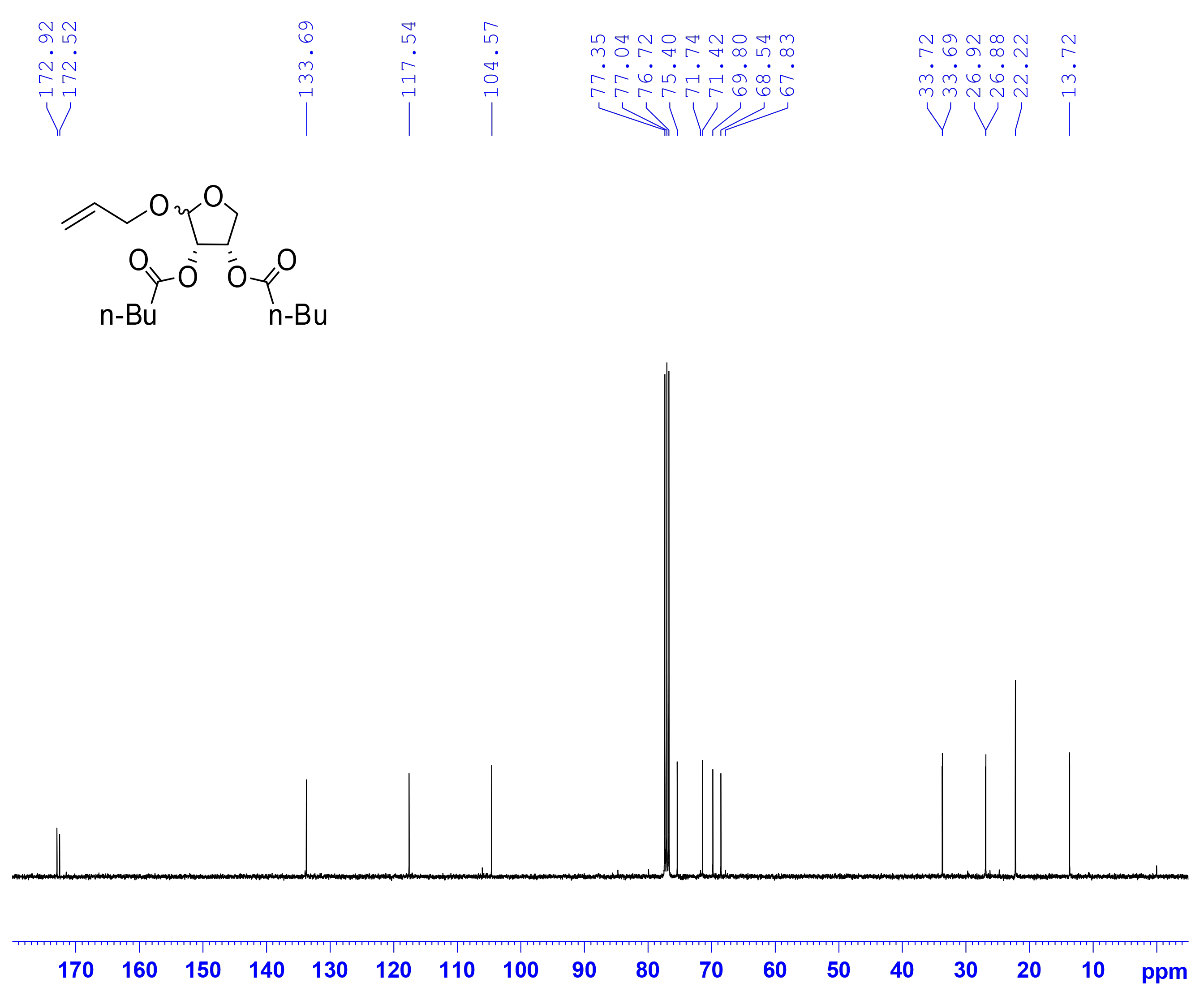

Supplement: Supplementary file 2 [file DataSheet1.ZIP › Supplementary Figure 15. 13C-NMR Allyloxy-2-valeric acid-3,4-tetrahydrofuran diester.tif]

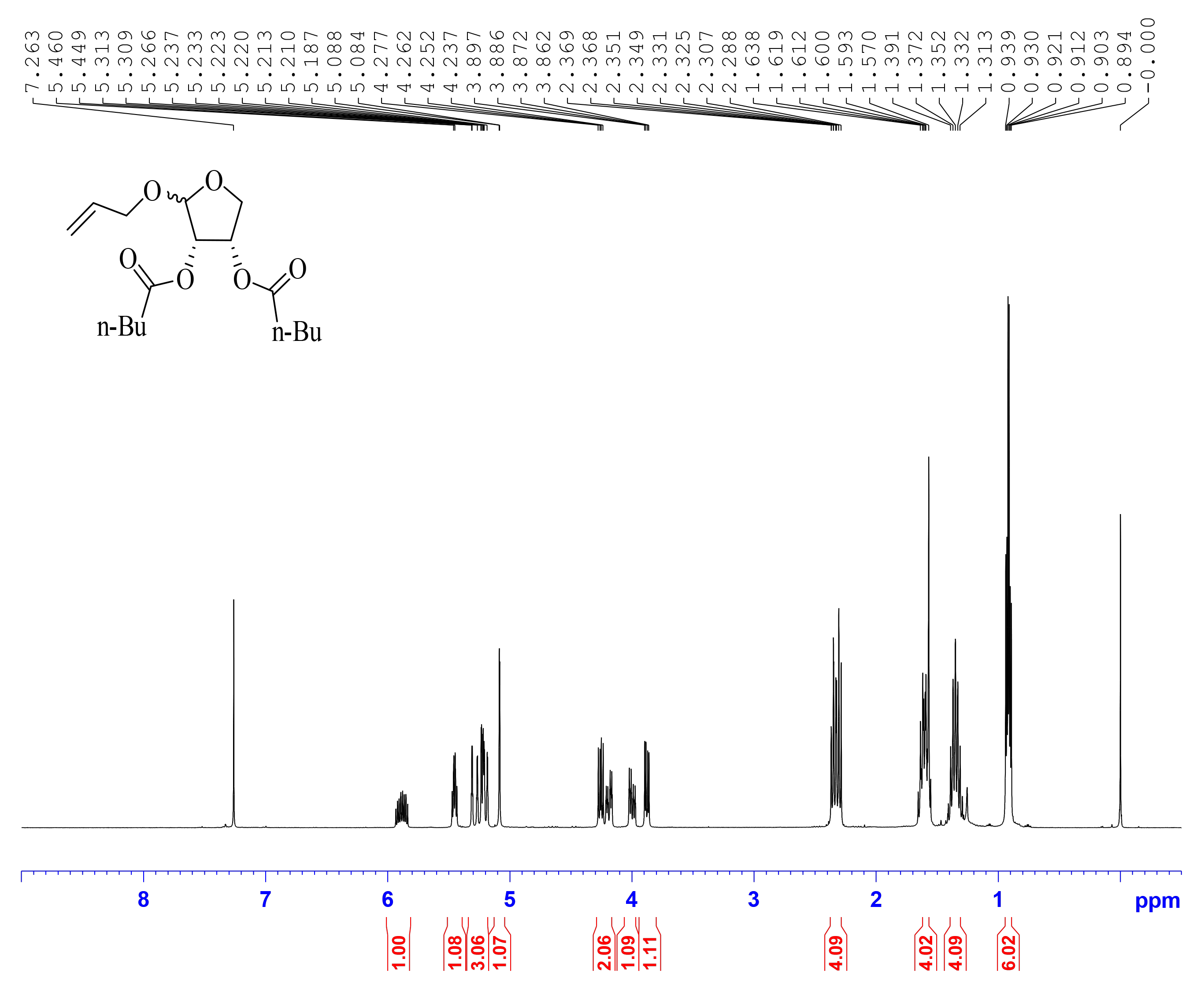

Supplement: Supplementary file 2 [file DataSheet1.ZIP › Supplementary Figure 16. 1H-NMR Allyloxy-2-valeric acid-3,4-tetrahydrofuran diester.tif]

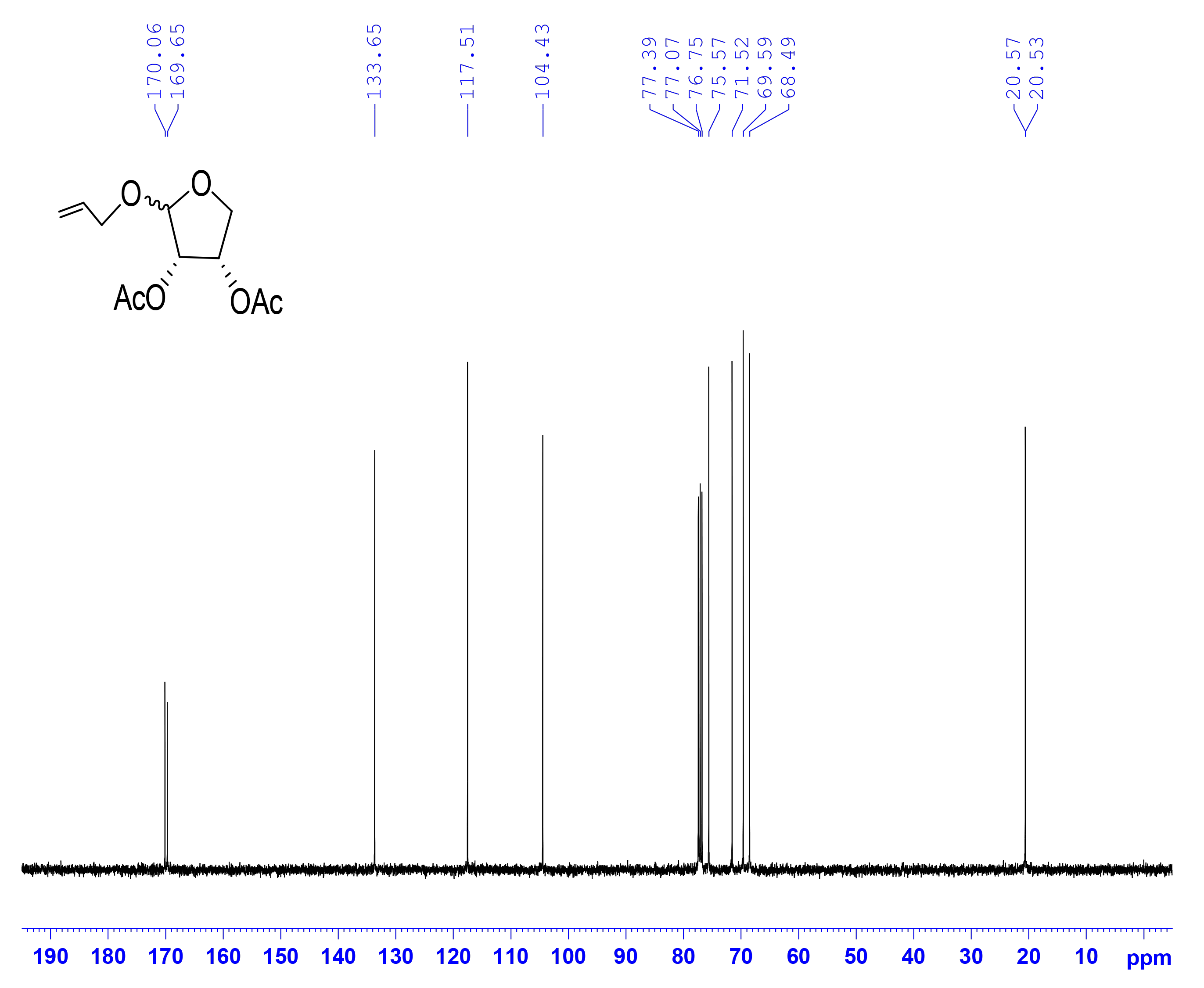

Supplement: Supplementary file 2 [file DataSheet1.ZIP › Supplementary Figure 17. 13C-NMR Allyloxy-2-acetic acid-3,4-tetrahydrofuran diester.tif]

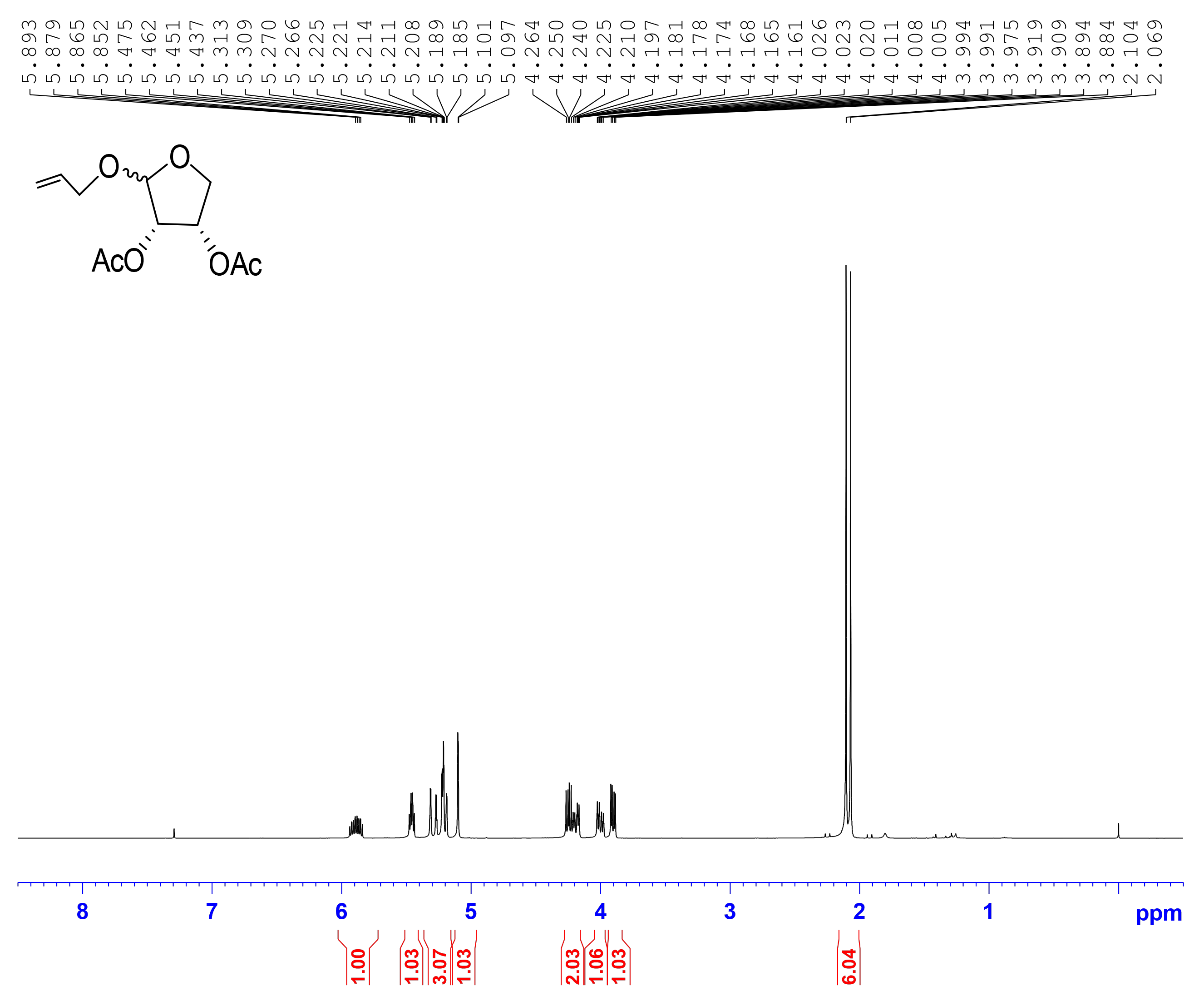

Supplement: Supplementary file 2 [file DataSheet1.ZIP › Supplementary Figure 18. 1H-NMR Allyloxy-2-acetic acid-3,4-tetrahydrofuran diester.tif]

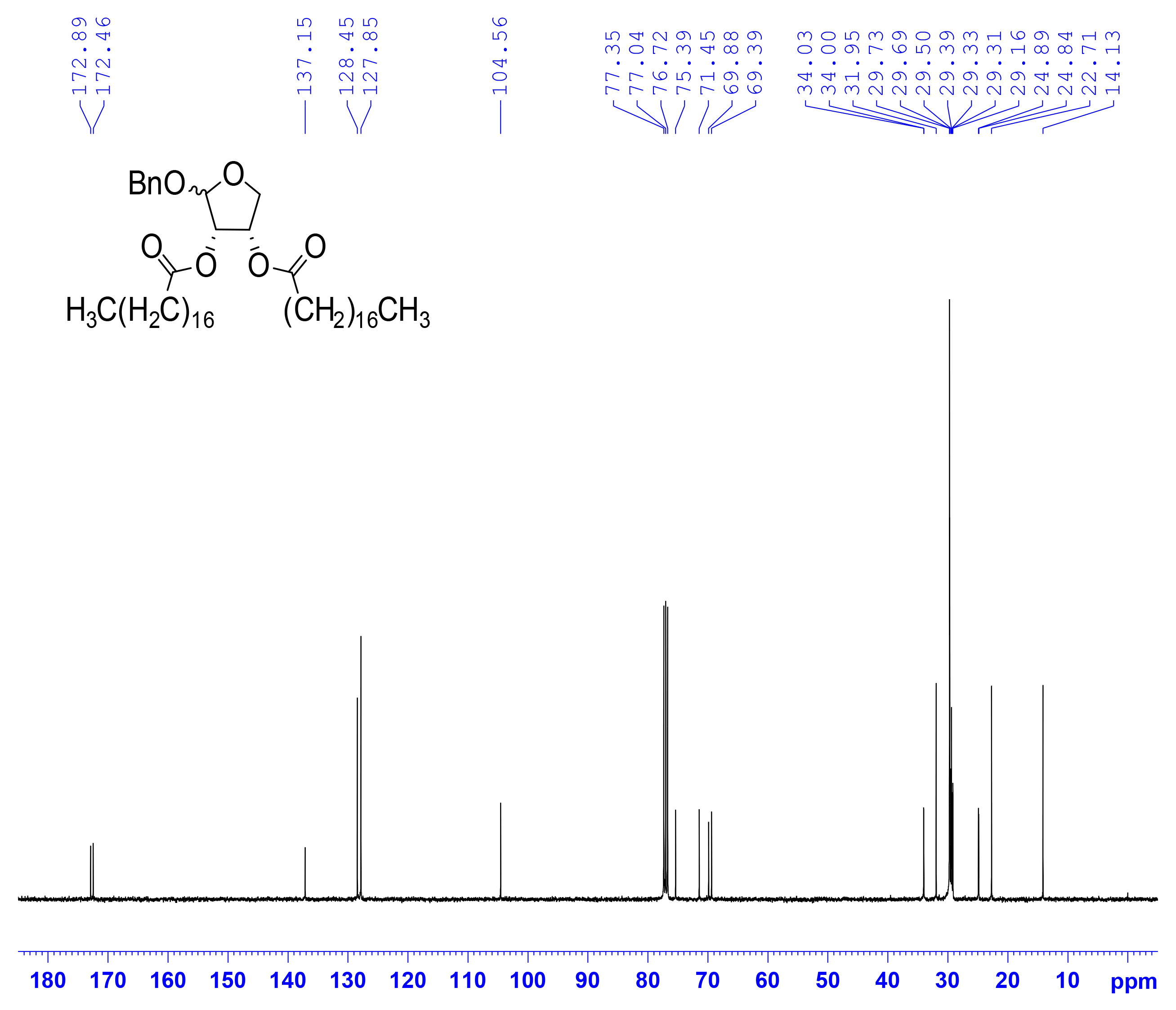

Supplement: Supplementary file 2 [file DataSheet1.ZIP › Supplementary Figure 19. 13C-NMR Benzyloxy-2-octadecarbonate-3,4-tetrahydrofuran diester.tif]

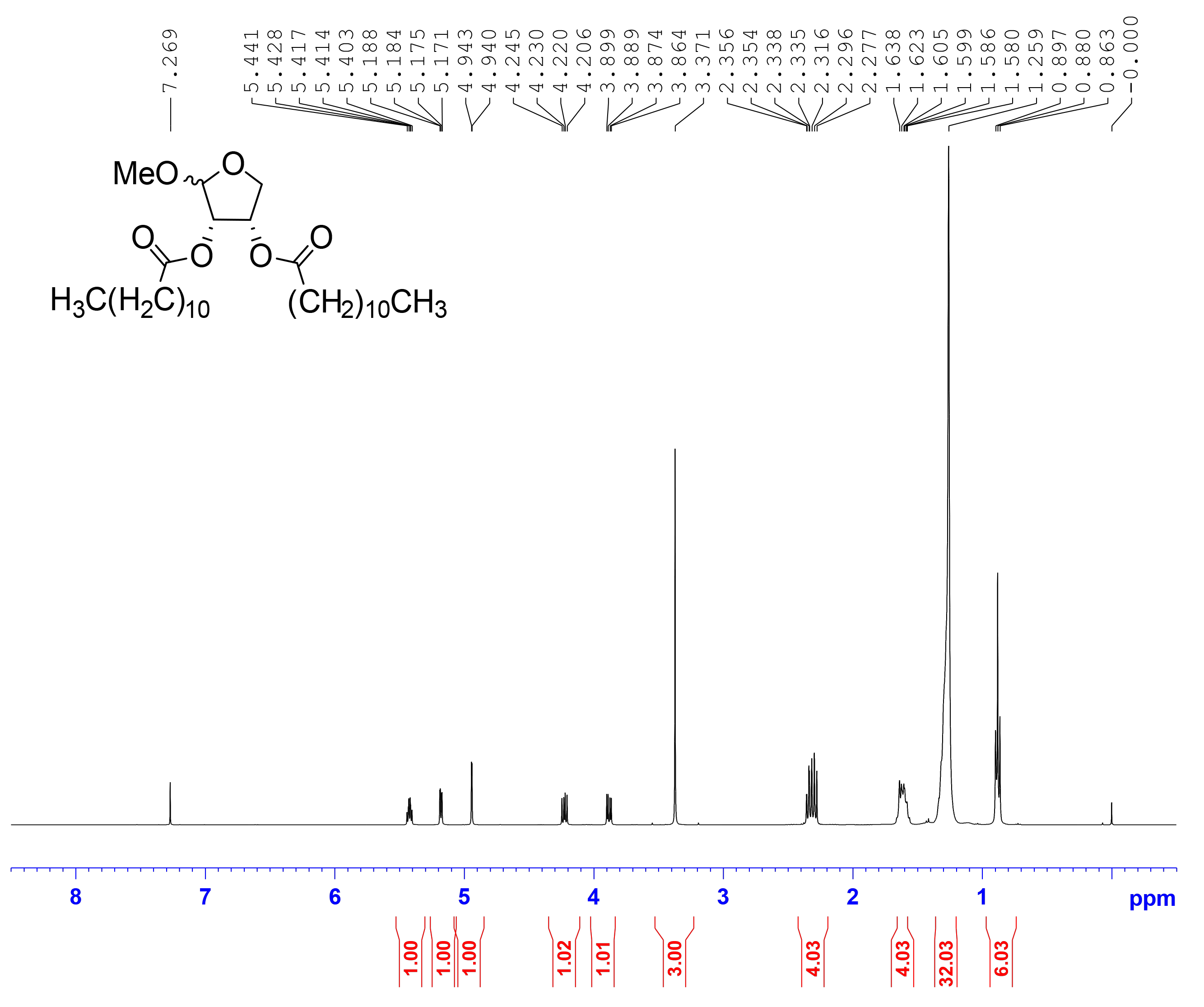

Supplement: Supplementary file 2 [file DataSheet1.ZIP › Supplementary Figure 2. 1H-NMR Methoxy-2-dodecarbonate-3,4-tetrahydrofuran diester.tif]

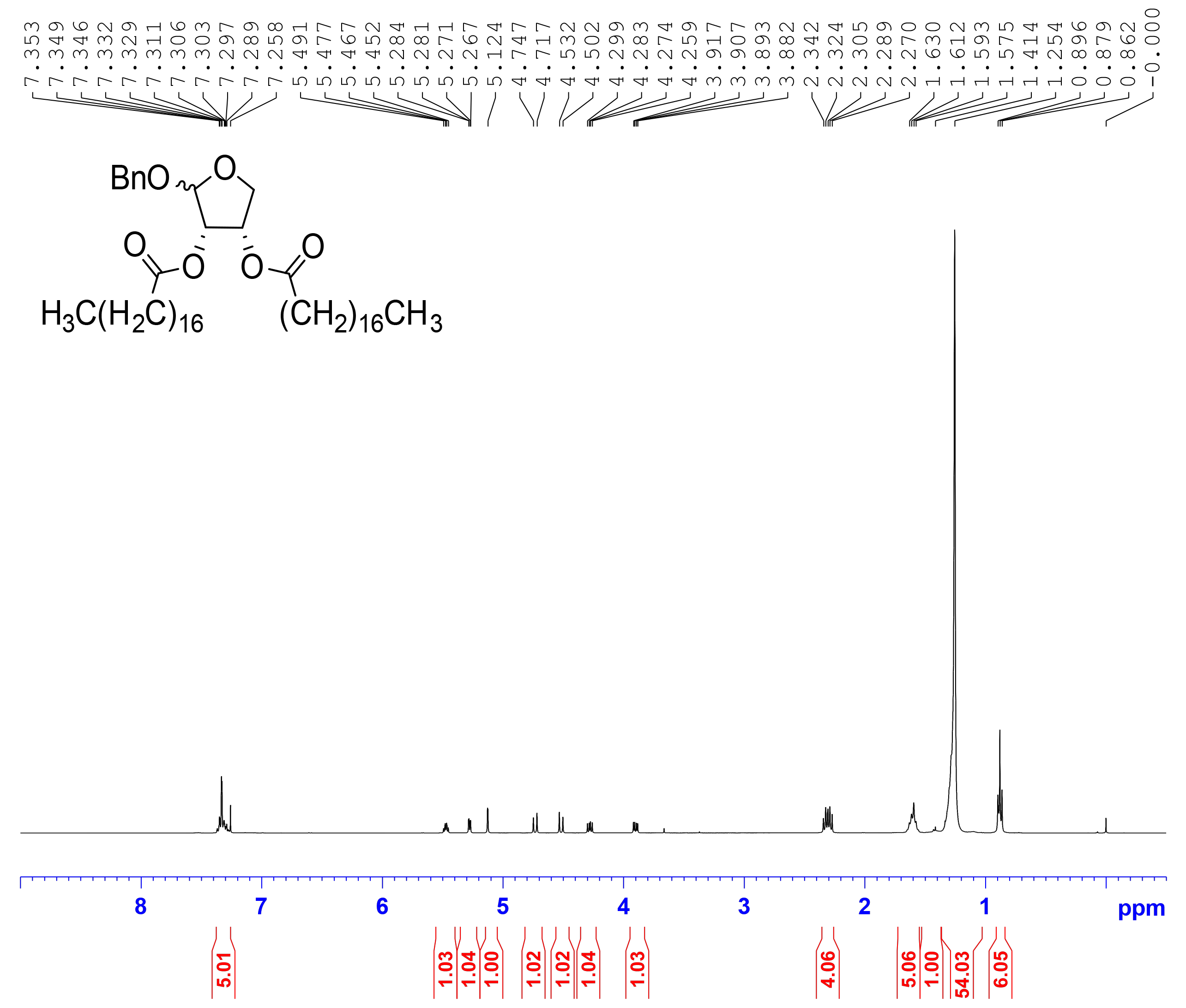

Supplement: Supplementary file 2 [file DataSheet1.ZIP › Supplementary Figure 20. 1H-NMR Benzyloxy-2-octadecarbonate-3,4-tetrahydrofuran diester.tif]

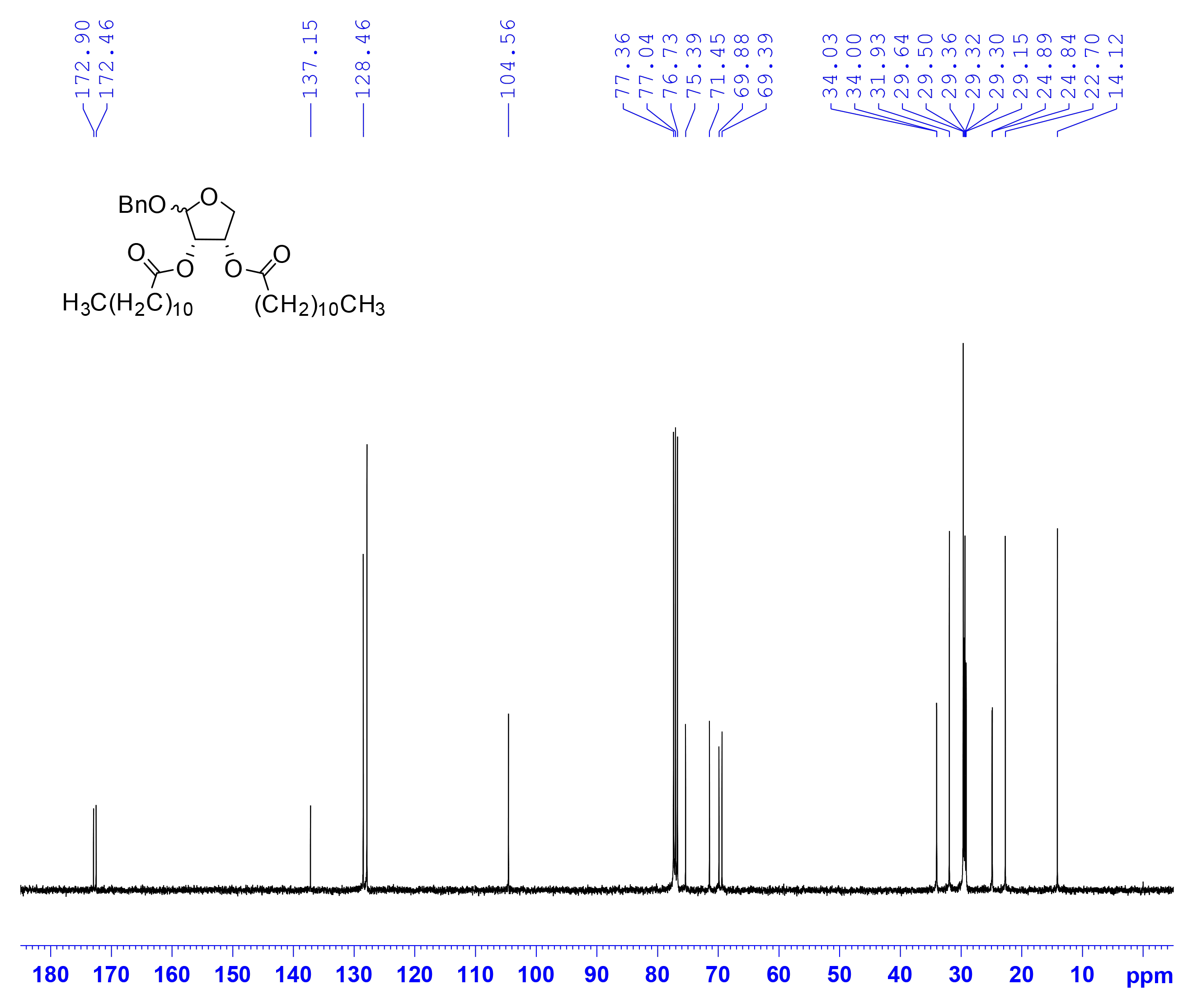

Supplement: Supplementary file 2 [file DataSheet1.ZIP › Supplementary Figure 21. 13C-NMR Benzyloxy-2-dodecarbonate-3,4-tetrahydrofuran diester.tif]

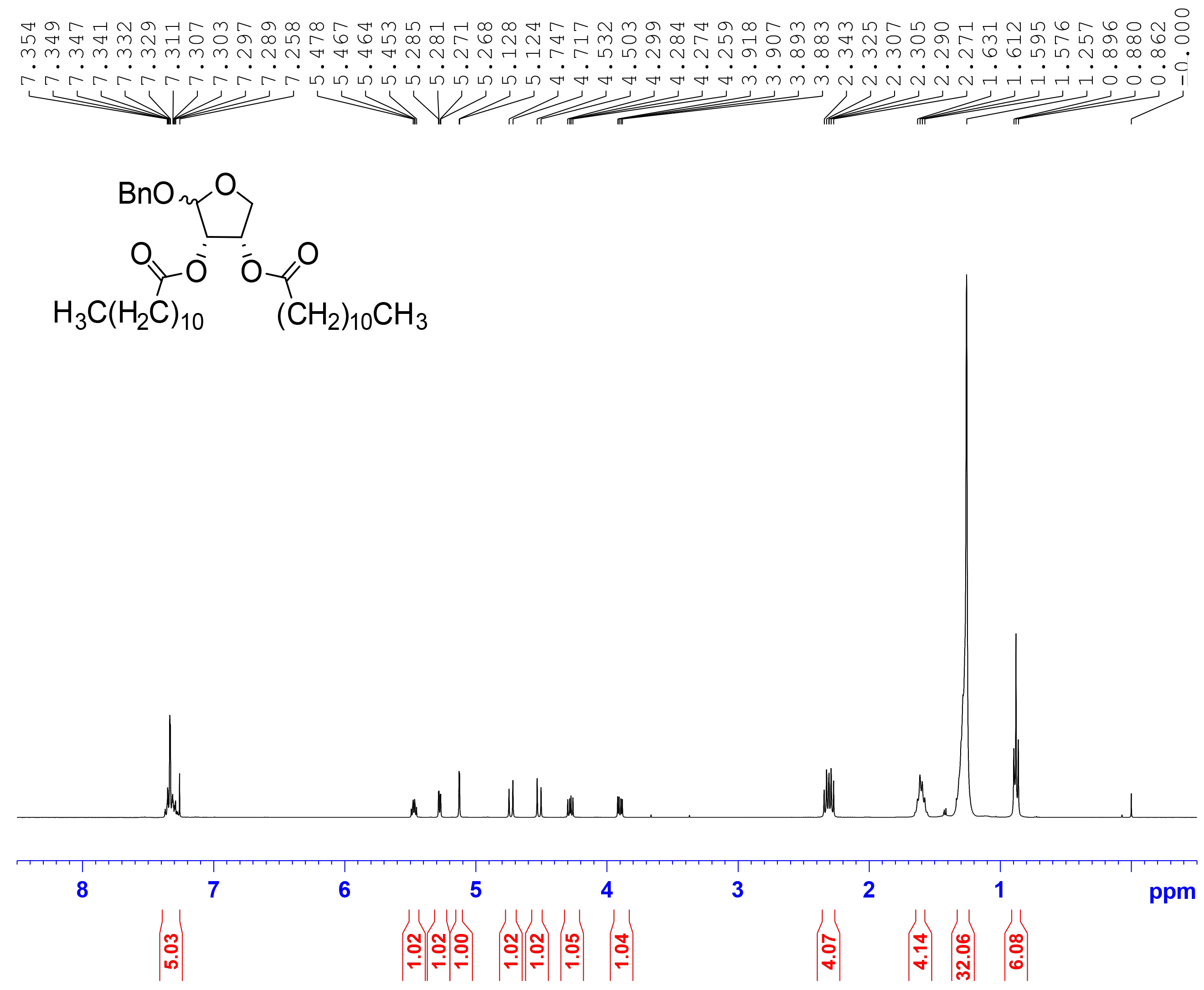

Supplement: Supplementary file 2 [file DataSheet1.ZIP › Supplementary Figure 22. 1H-NMR Benzyloxy-2-dodecarbonate-3,4-tetrahydrofuran diester.tif]

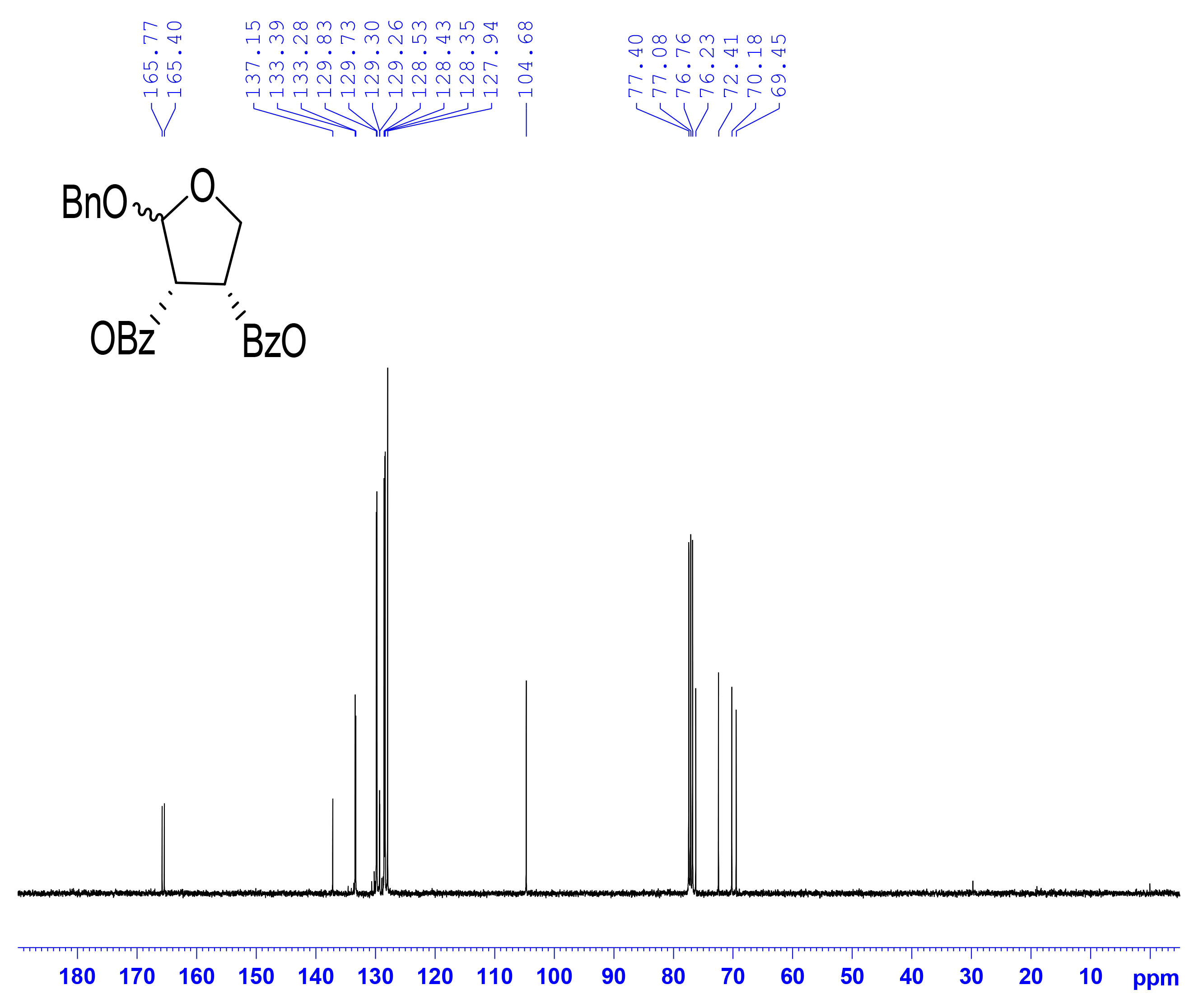

Supplement: Supplementary file 2 [file DataSheet1.ZIP › Supplementary Figure 23. 13C-NMR Benzyloxy-2-benzoic acid-3,4-tetrahydrofuran diester.tif]

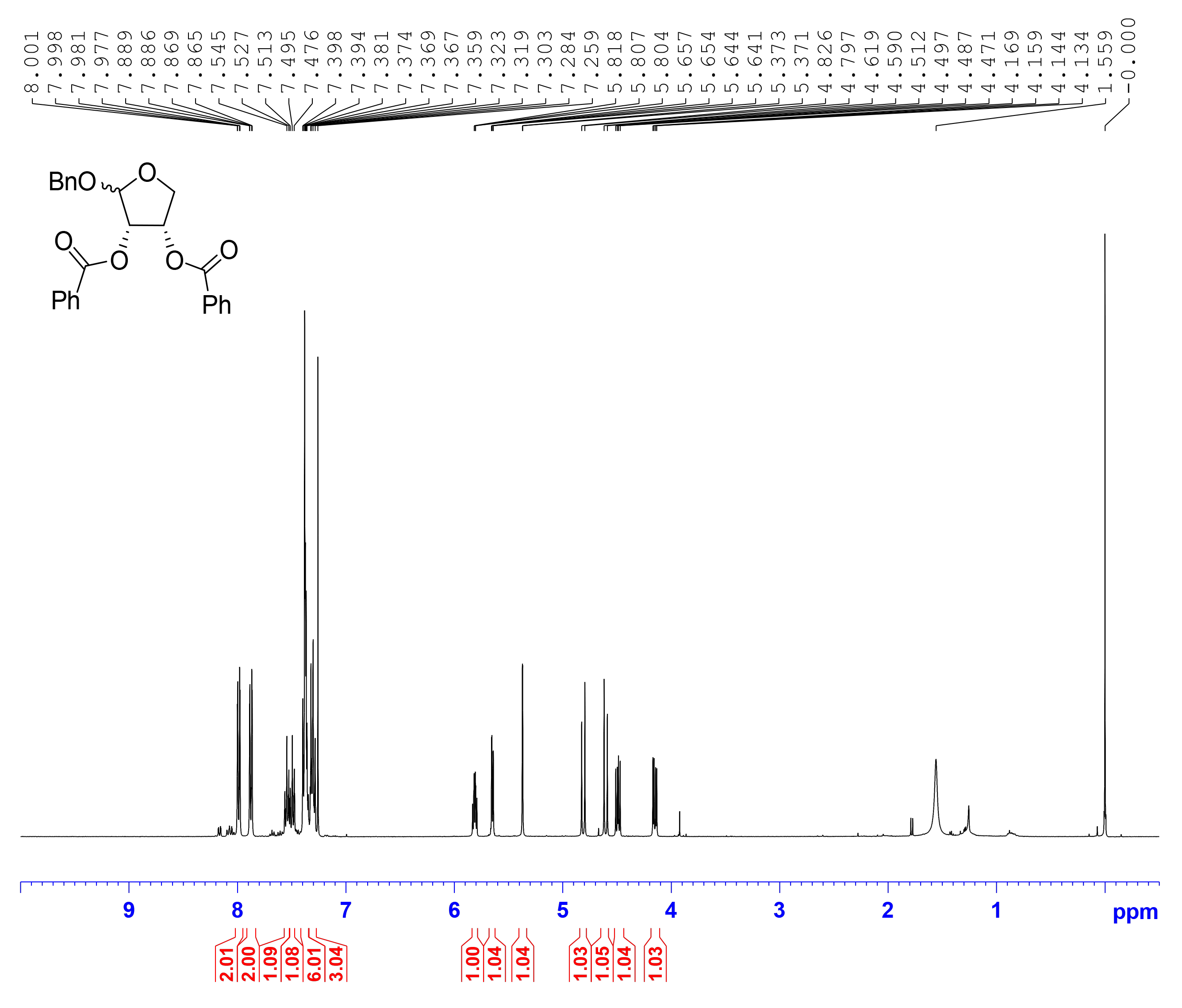

Supplement: Supplementary file 2 [file DataSheet1.ZIP › Supplementary Figure 24. 1H-NMR Benzyloxy-2-benzoic acid-3,4-tetrahydrofuran diester.tif]

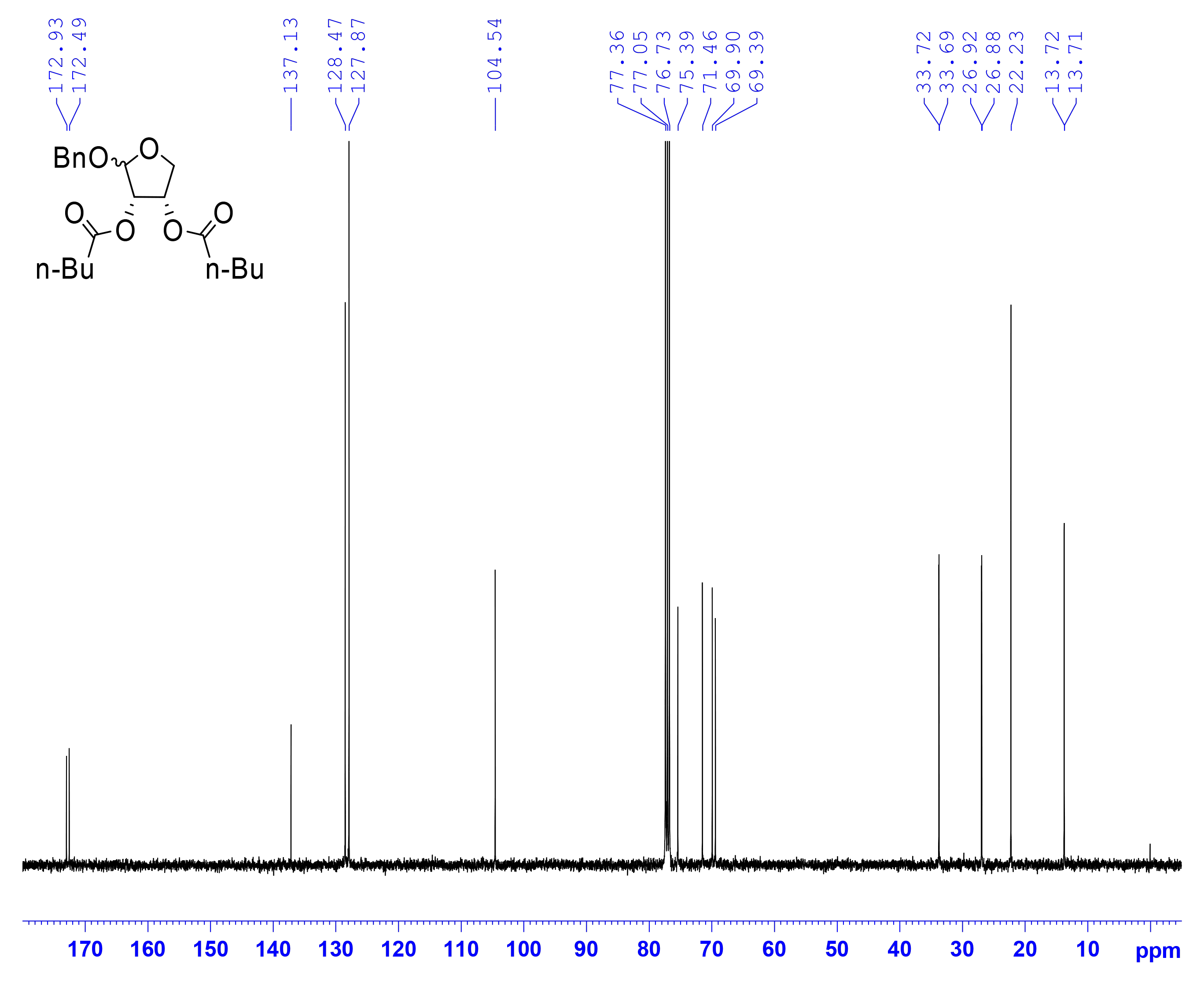

Supplement: Supplementary file 2 [file DataSheet1.ZIP › Supplementary Figure 25. 13C-NMR Benzyloxy-2- valeric acid-3,4-tetrahydrofuran diester.tif]

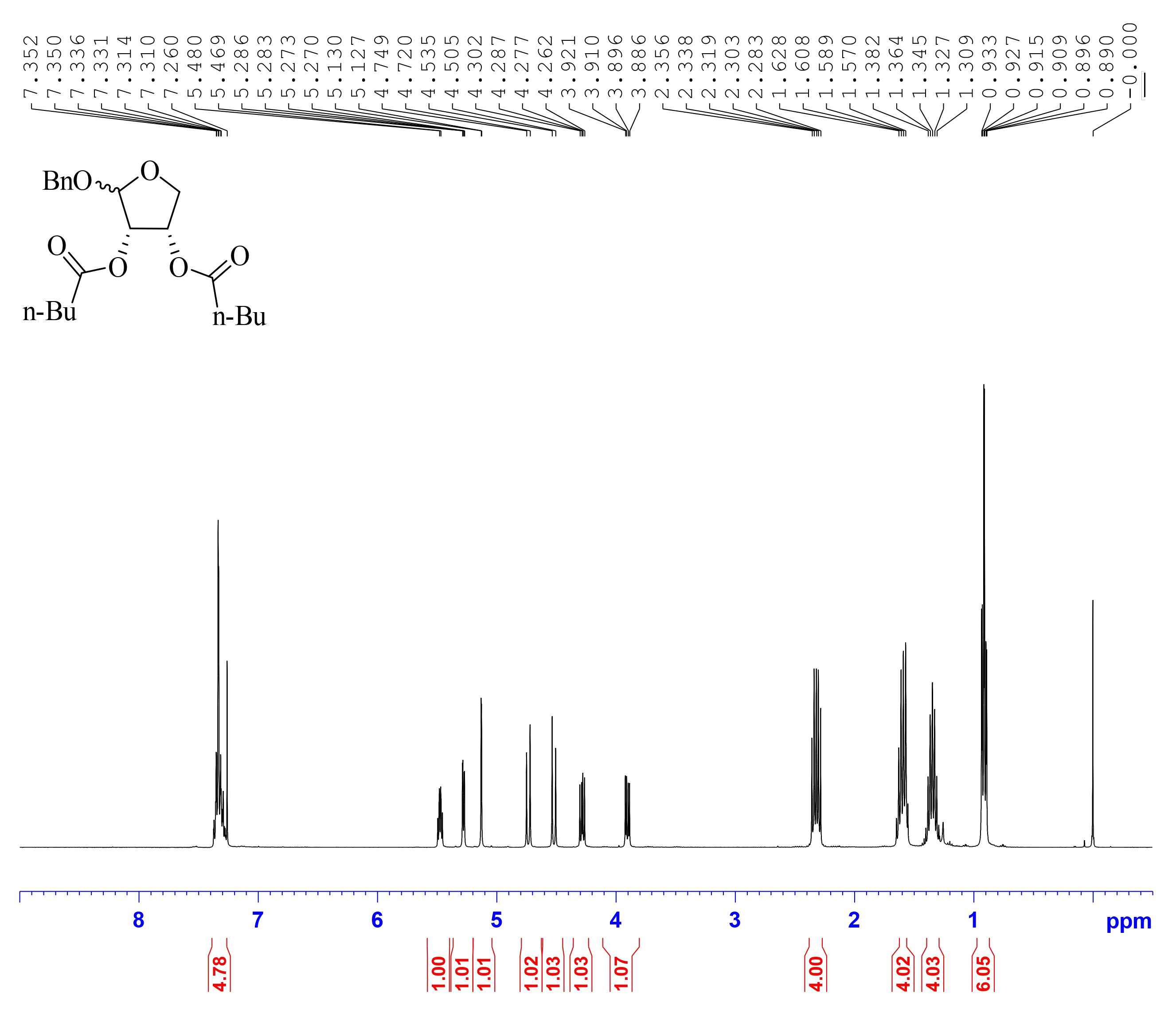

Supplement: Supplementary file 2 [file DataSheet1.ZIP › Supplementary Figure 26. 1H-NMR Benzyloxy-2- valeric acid-3,4-tetrahydrofuran diester.tif]

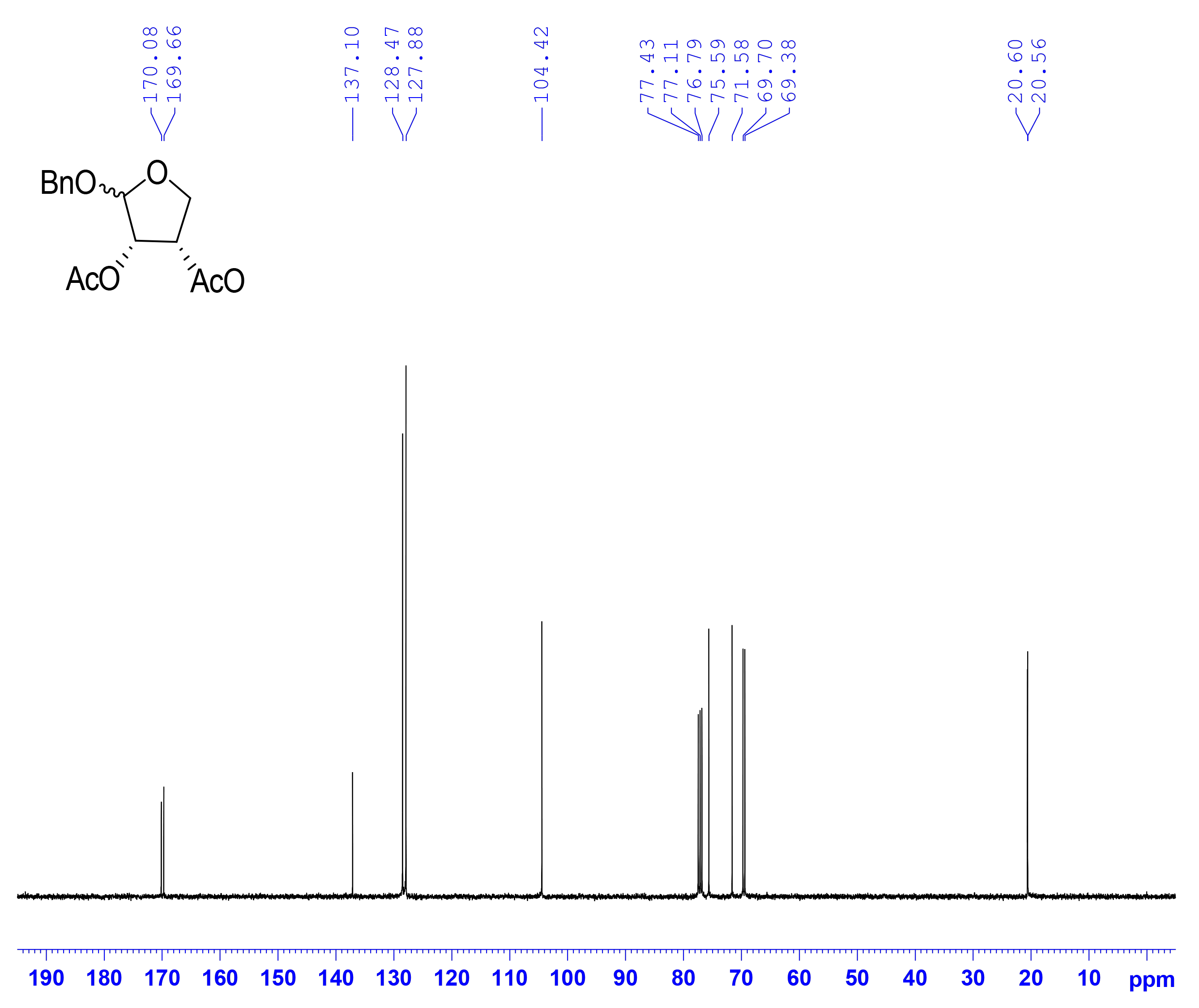

Supplement: Supplementary file 2 [file DataSheet1.ZIP › Supplementary Figure 27. 13C-NMR Benzyloxy-2-acetic acid-3,4-tetrahydrofuran diester.tif]

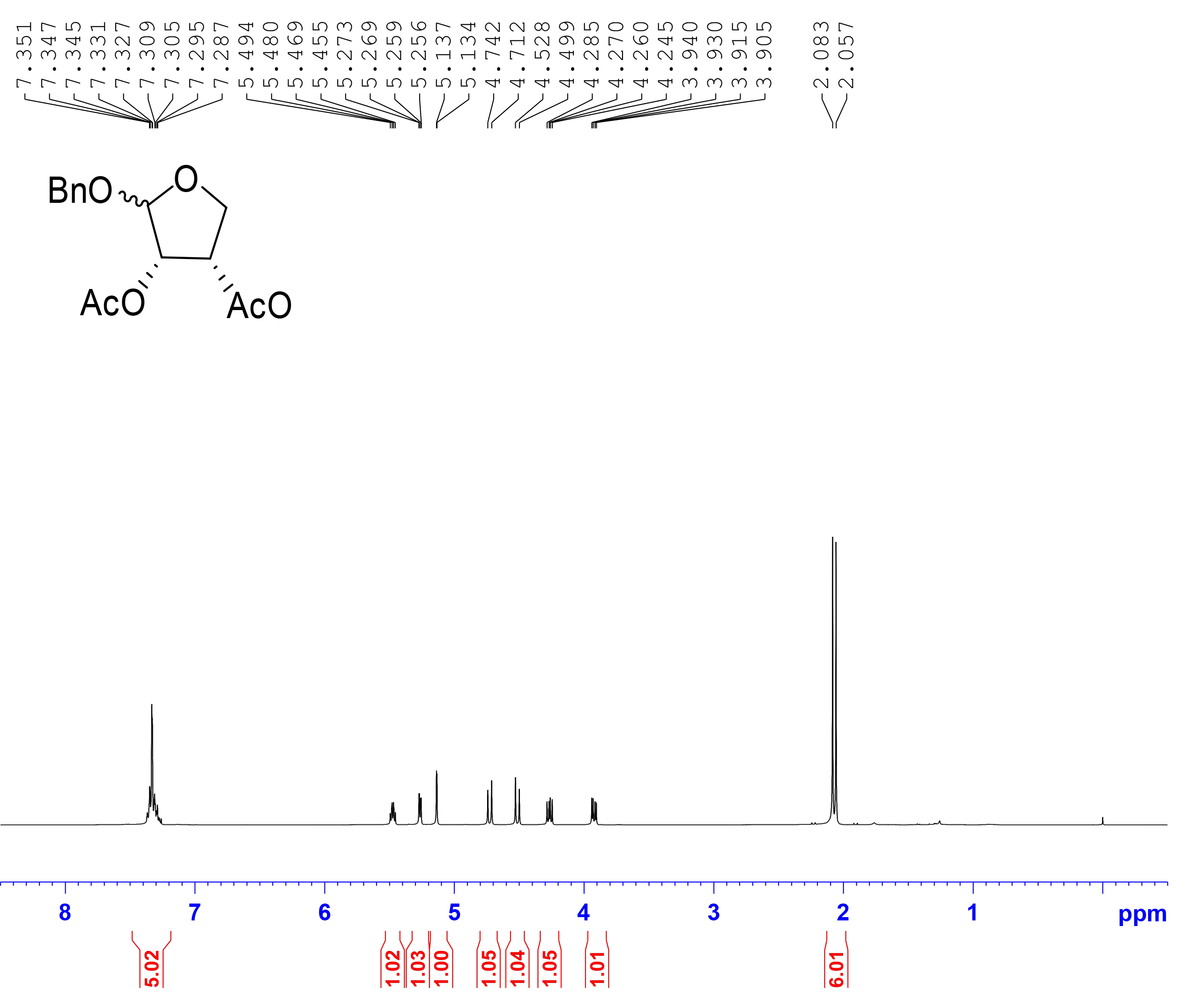

Supplement: Supplementary file 2 [file DataSheet1.ZIP › Supplementary Figure 28. 1H-NMR Benzyloxy-2-acetic acid-3,4-tetrahydrofuran diester.tif]

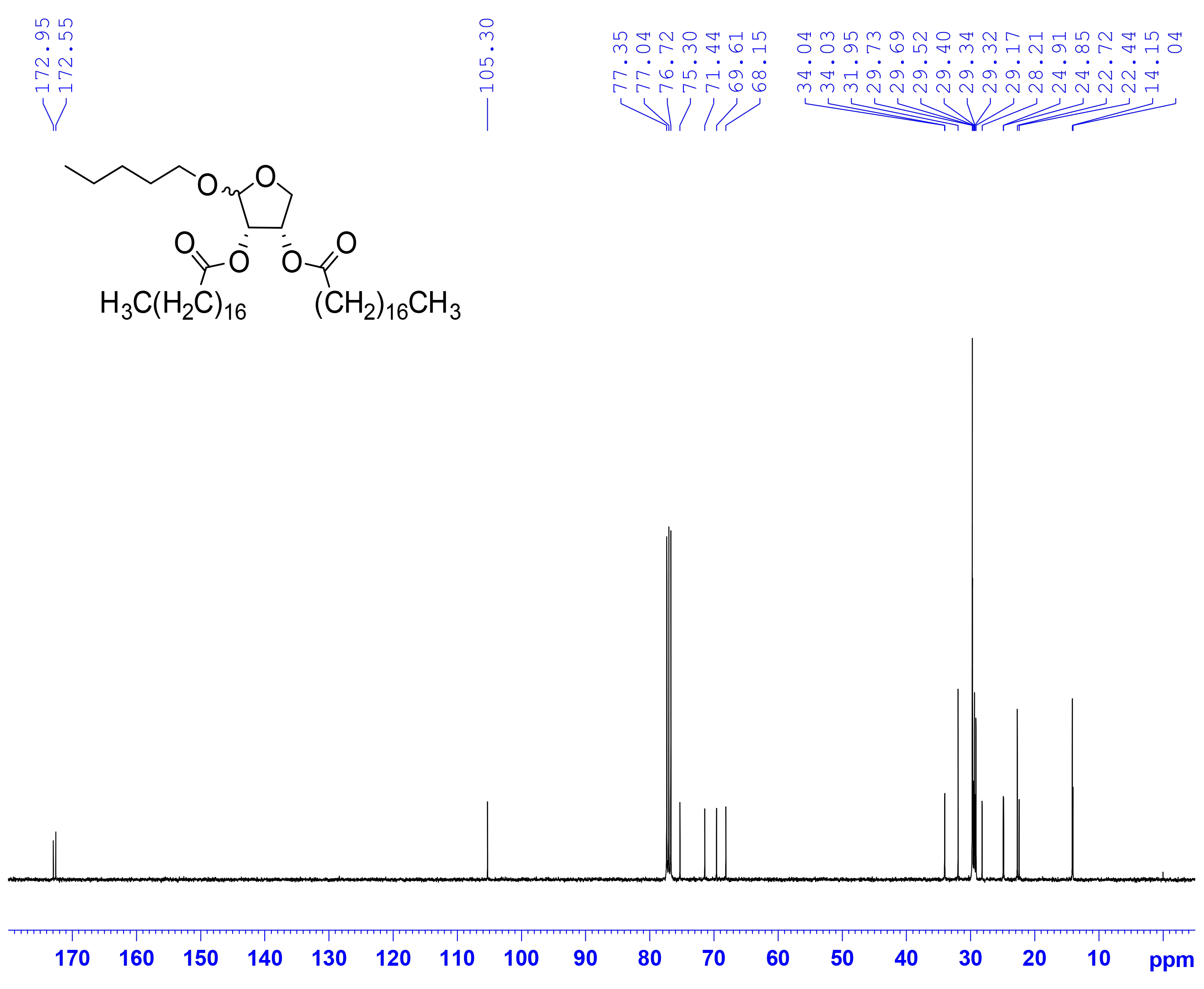

Supplement: Supplementary file 2 [file DataSheet1.ZIP › Supplementary Figure 29. 13C-NMR Pentoxy-2- octadecarbonate-3,4-tetrahydrofuran diester.tif]

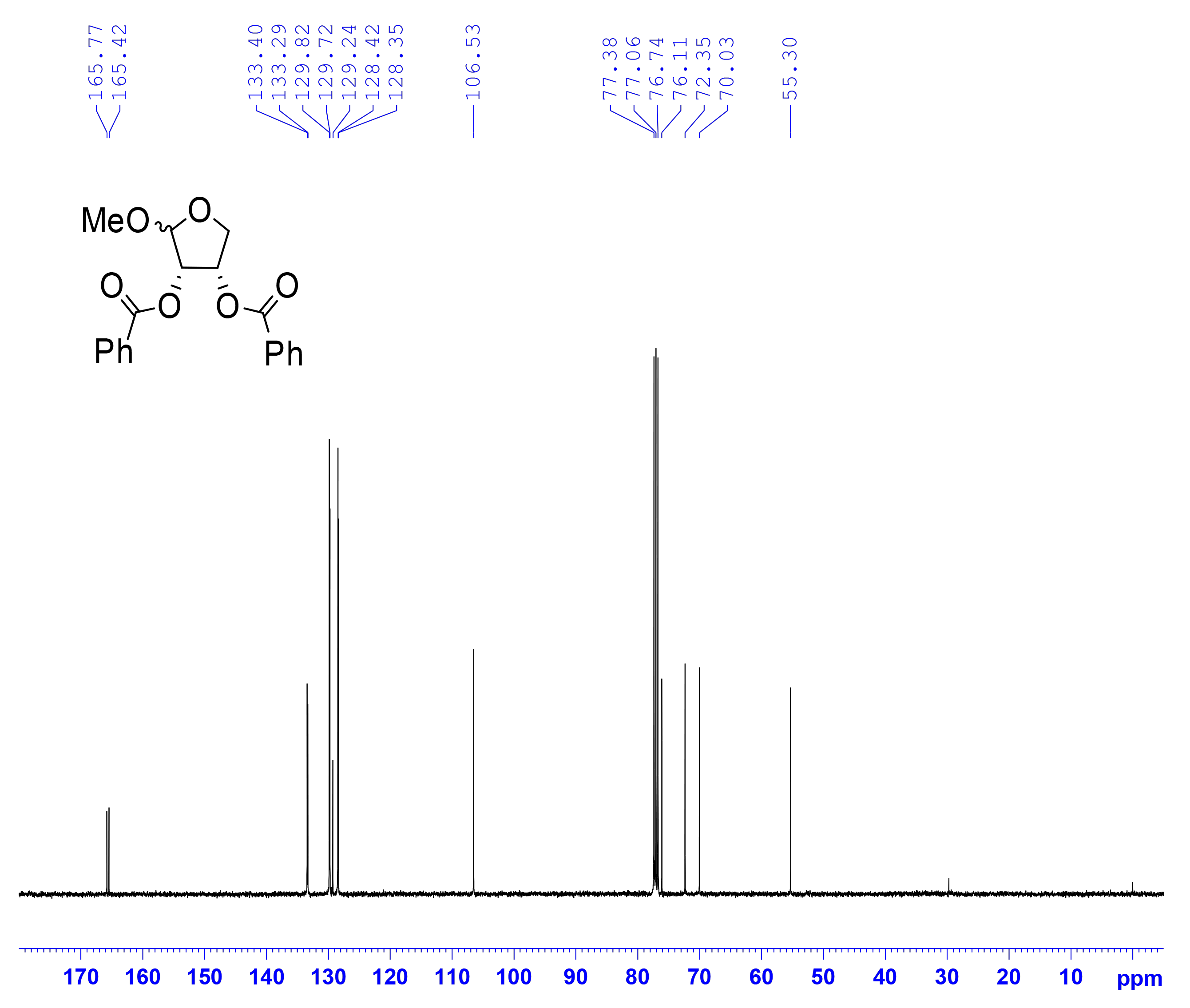

Supplement: Supplementary file 2 [file DataSheet1.ZIP › Supplementary Figure 3. 13C-NMR Methoxy-2-benzoic acid-3,4-tetrahydrofuran diester.tif]

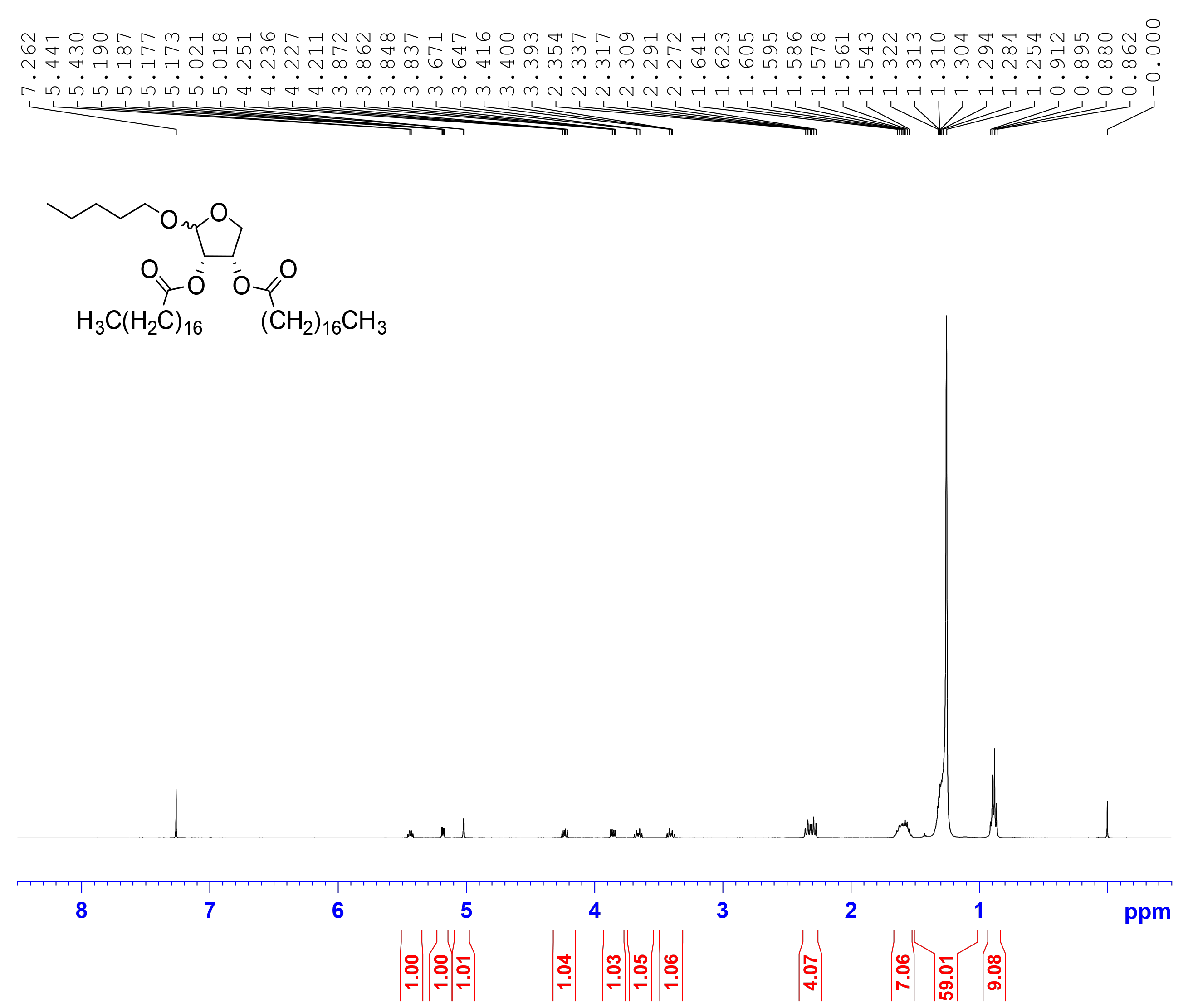

Supplement: Supplementary file 2 [file DataSheet1.ZIP › Supplementary Figure 30. 1H-NMR Pentoxy-2- octadecarbonate-3,4-tetrahydrofuran diester.tif]

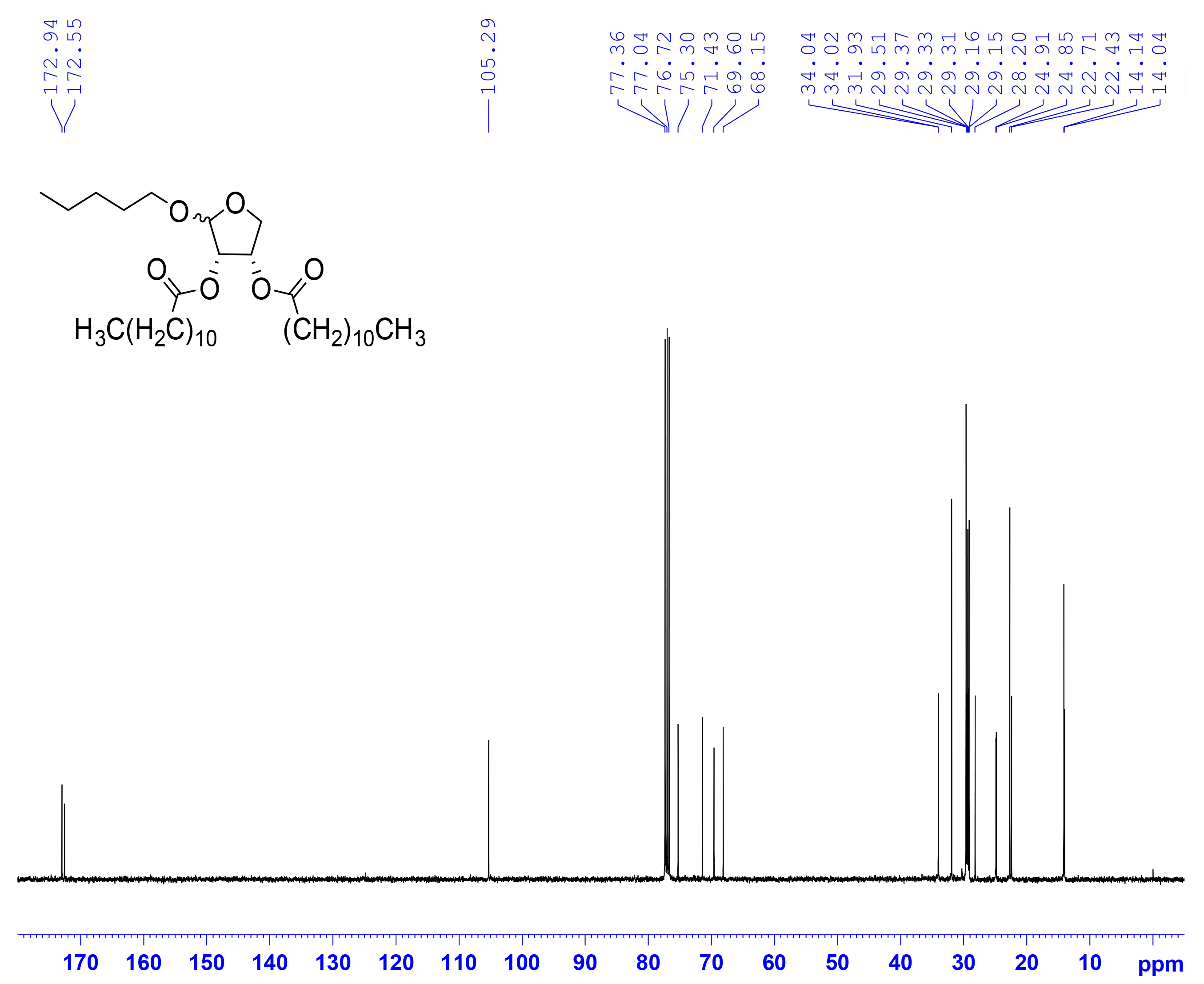

Supplement: Supplementary file 2 [file DataSheet1.ZIP › Supplementary Figure 31. 13C-NMR Pentoxy-2- dodecarbonate-3,4-tetrahydrofuran diester.tif]

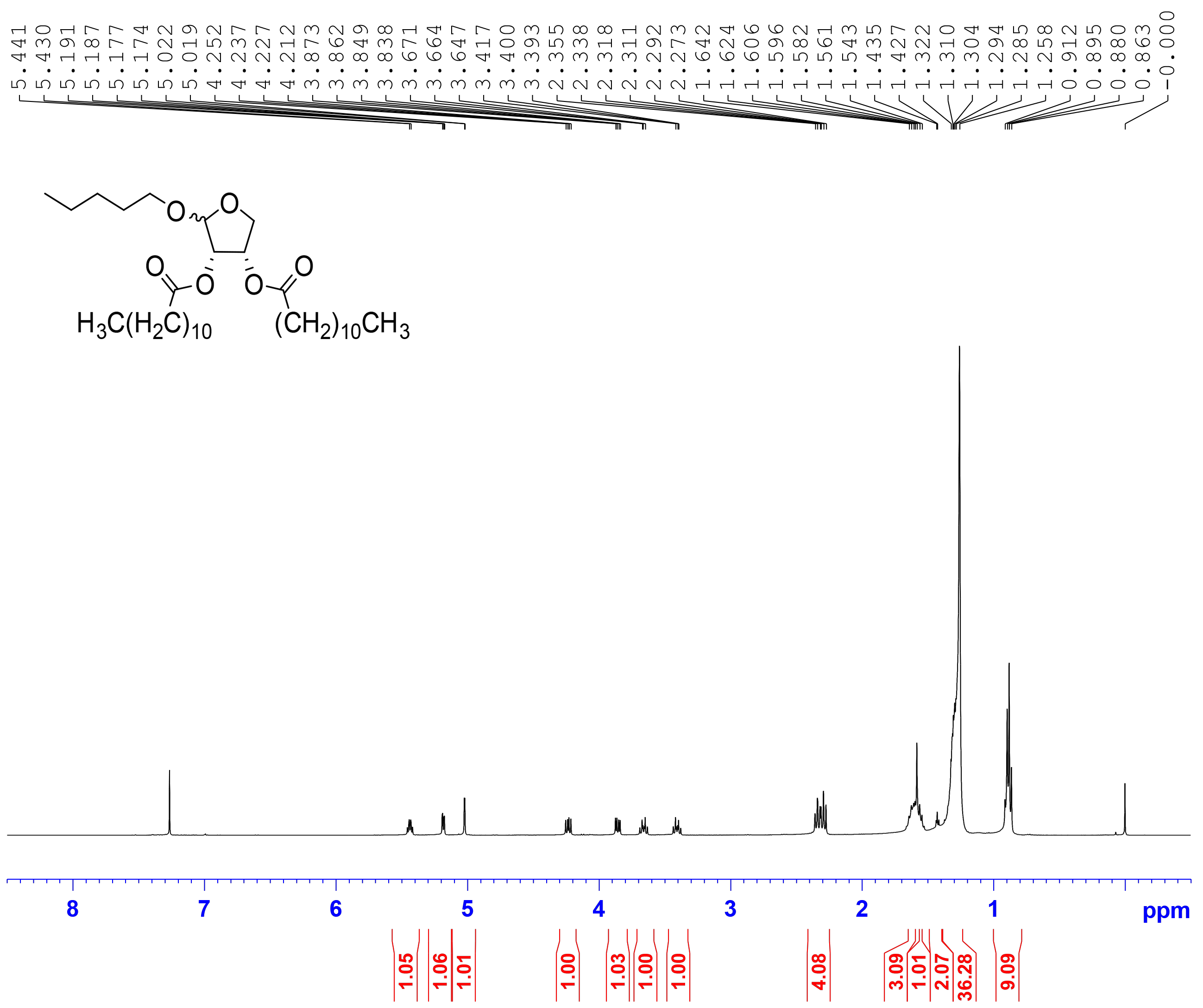

Supplement: Supplementary file 2 [file DataSheet1.ZIP › Supplementary Figure 32. 1H-NMR Pentoxy-2- dodecarbonate-3,4-tetrahydrofuran diester.tif]

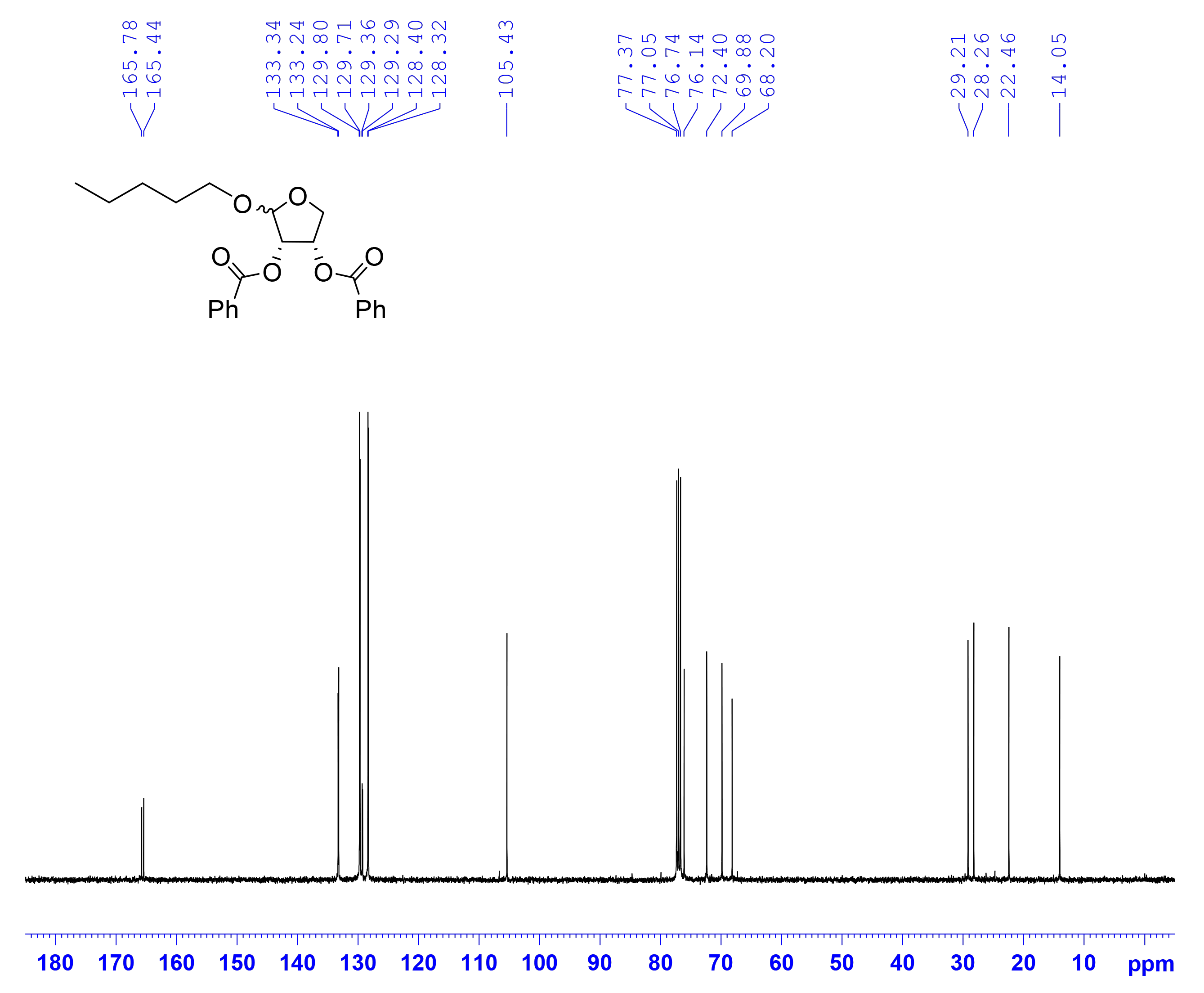

Supplement: Supplementary file 2 [file DataSheet1.ZIP › Supplementary Figure 33. 13C-NMR Pentoxy-2-benzoic acid-3,4-tetrahydrofuran diester.tif]

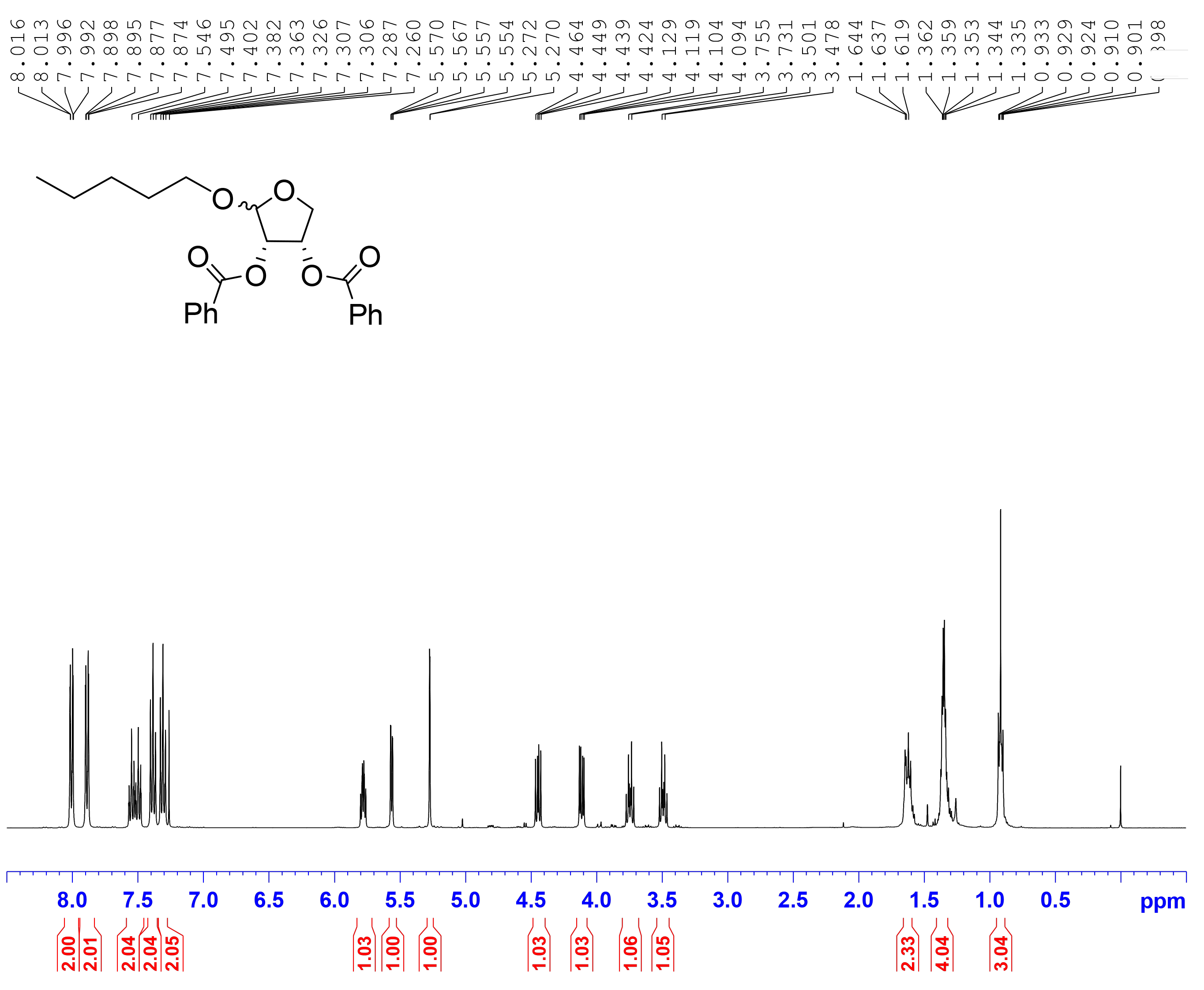

Supplement: Supplementary file 2 [file DataSheet1.ZIP › Supplementary Figure 34. 1H-NMR Pentoxy-2-benzoic acid-3,4-tetrahydrofuran diester.tif]

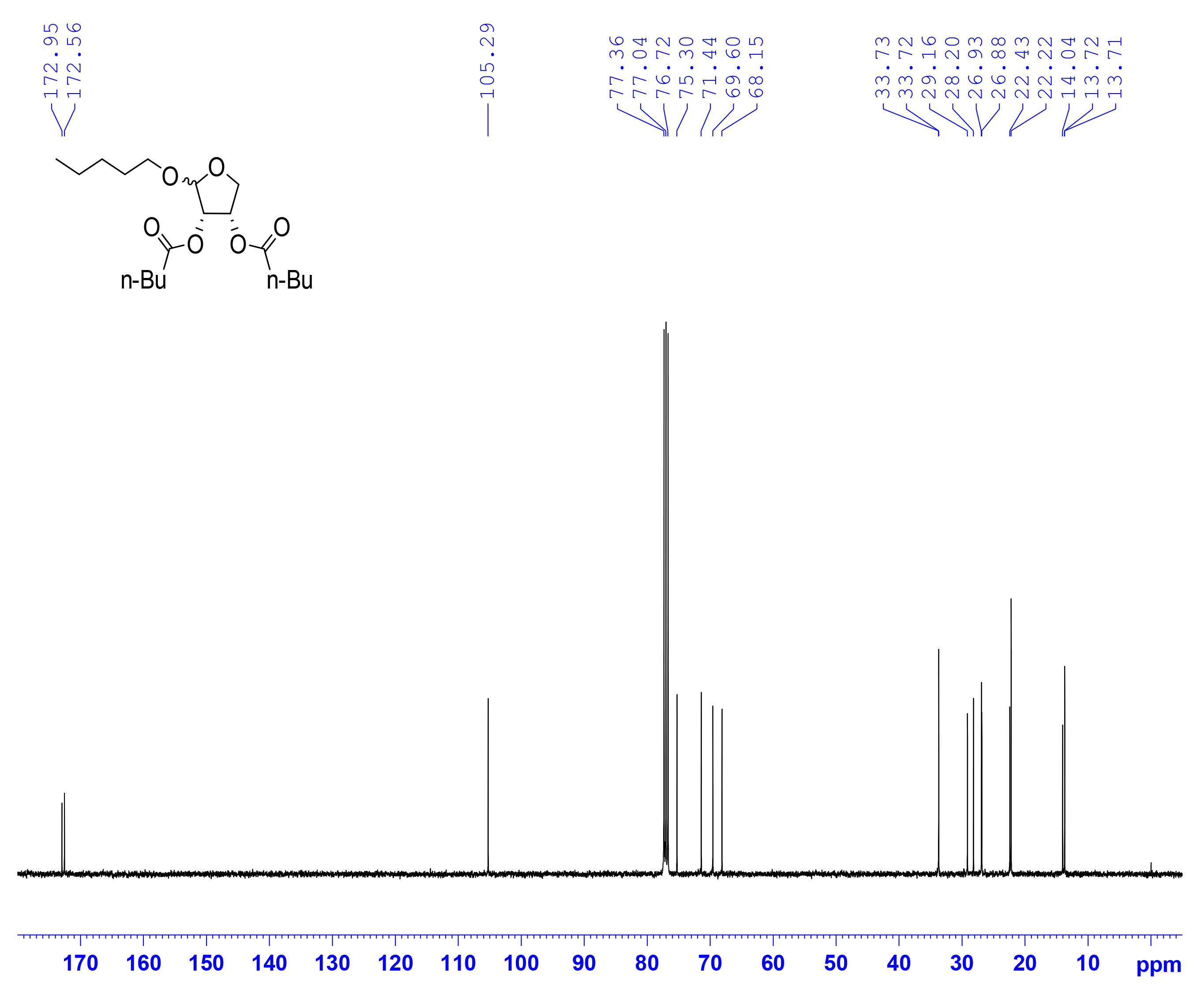

Supplement: Supplementary file 2 [file DataSheet1.ZIP › Supplementary Figure 35. 13C-NMR Pentoxy-2-valeric acid-3,4-tetrahydrofuran diester.tif]

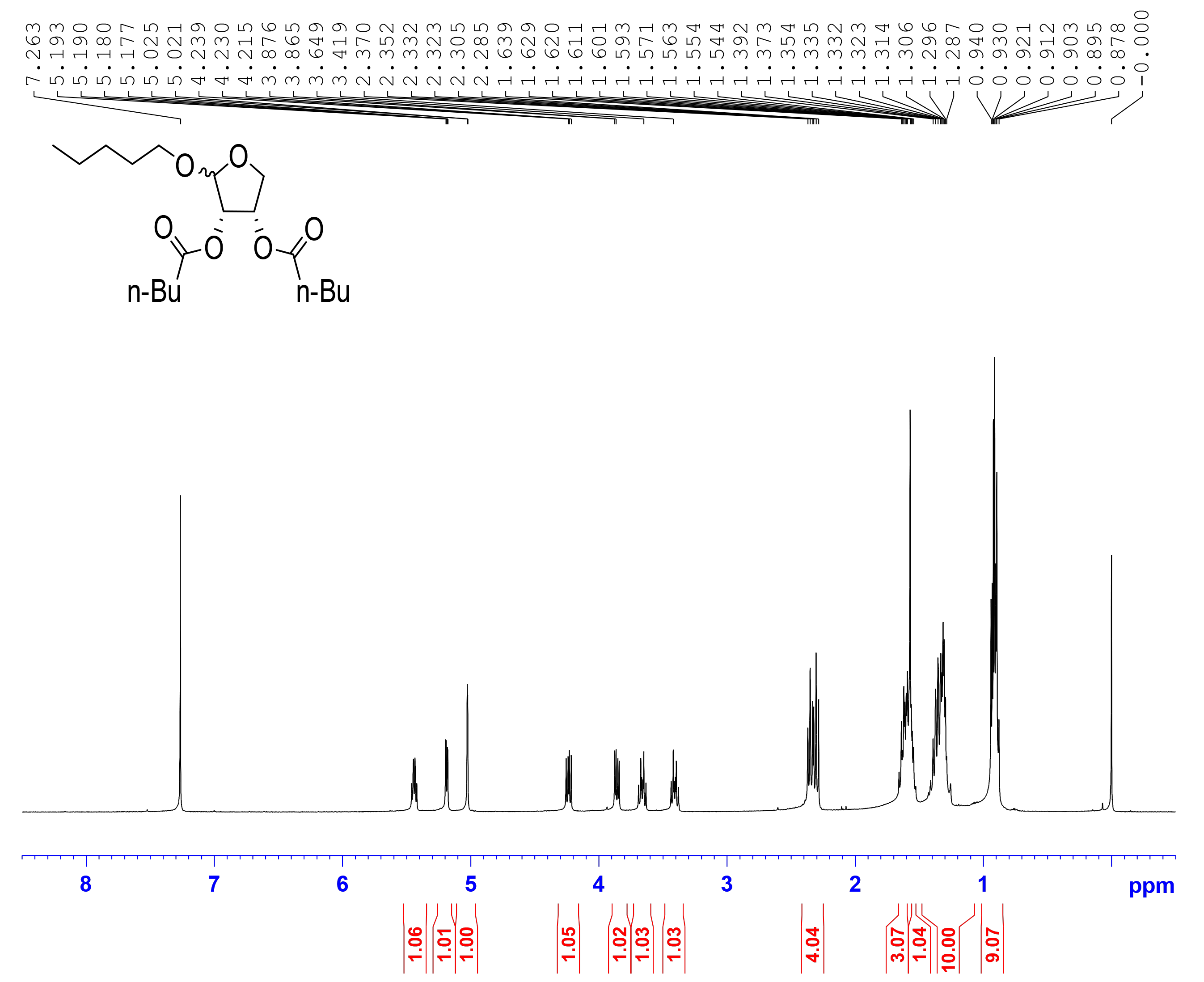

Supplement: Supplementary file 2 [file DataSheet1.ZIP › Supplementary Figure 36. 1H-NMR Pentoxy-2-valeric acid-3,4-tetrahydrofuran diester.tif]

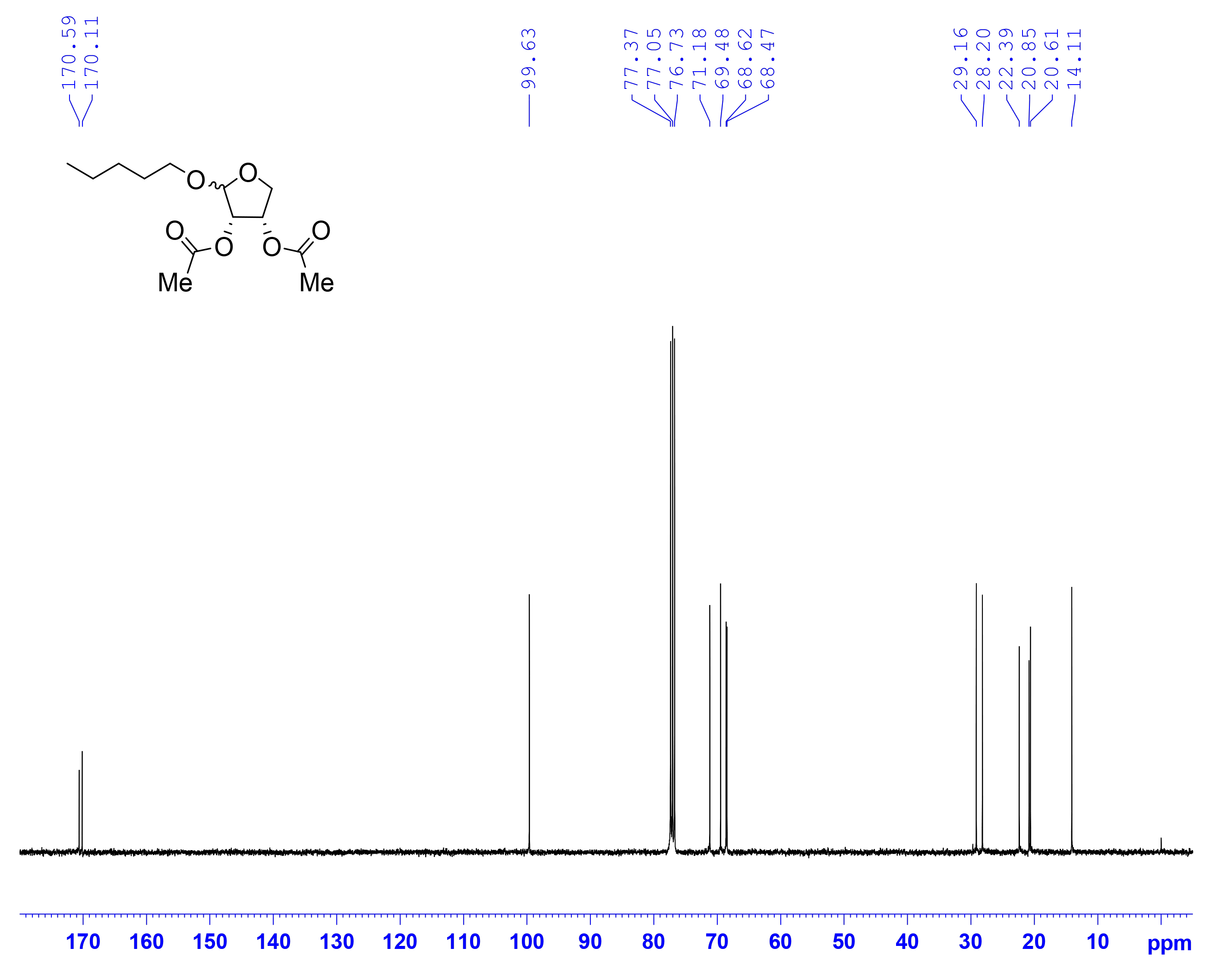

Supplement: Supplementary file 2 [file DataSheet1.ZIP › Supplementary Figure 37. 13C-NMR Pentoxy-2-acetic acid-3,4-tetrahydrofuran diester.tif]

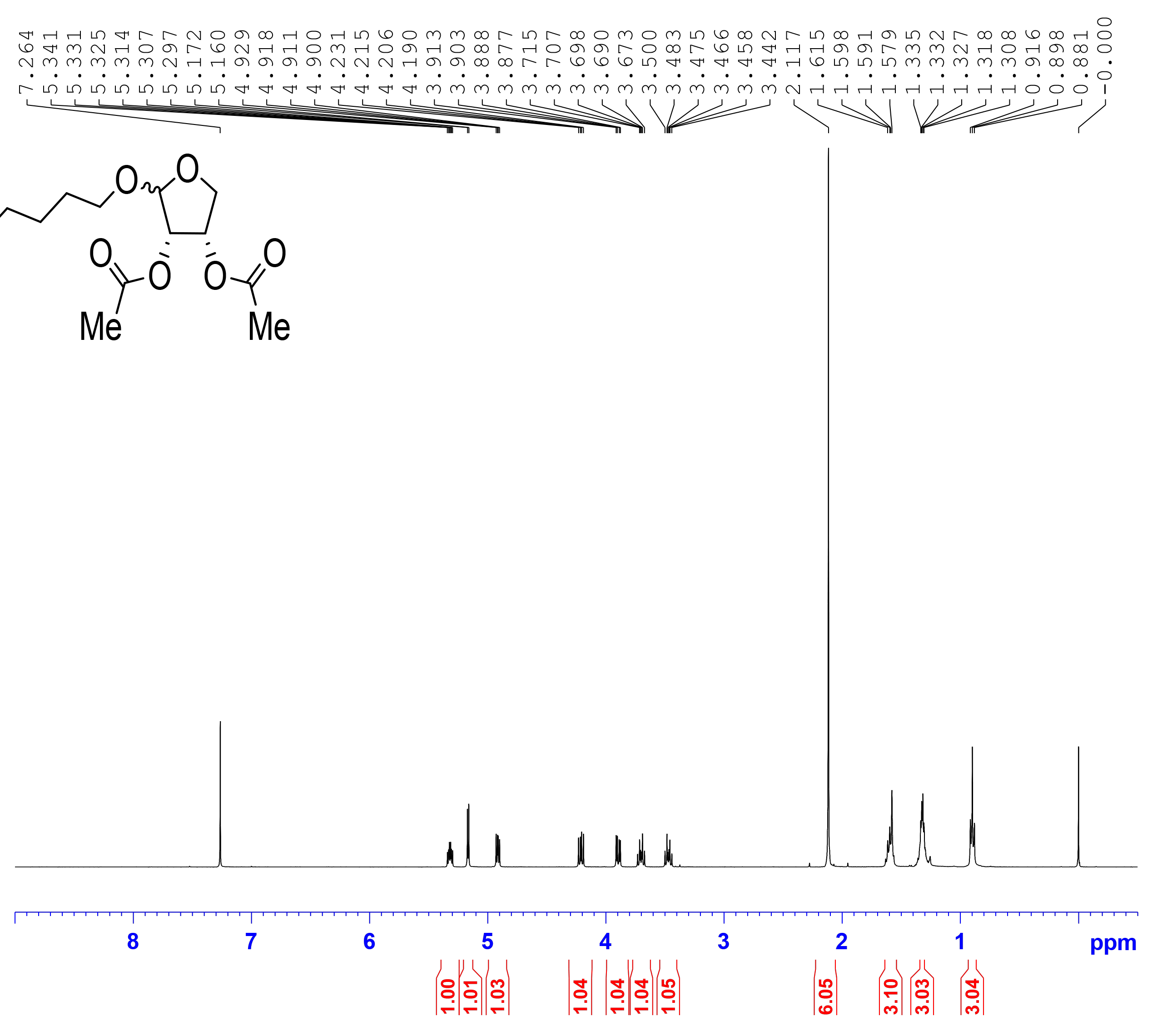

Supplement: Supplementary file 2 [file DataSheet1.ZIP › Supplementary Figure 38. 1H-NMR Pentoxy-2-acetic acid-3,4-tetrahydrofuran diester.tif]

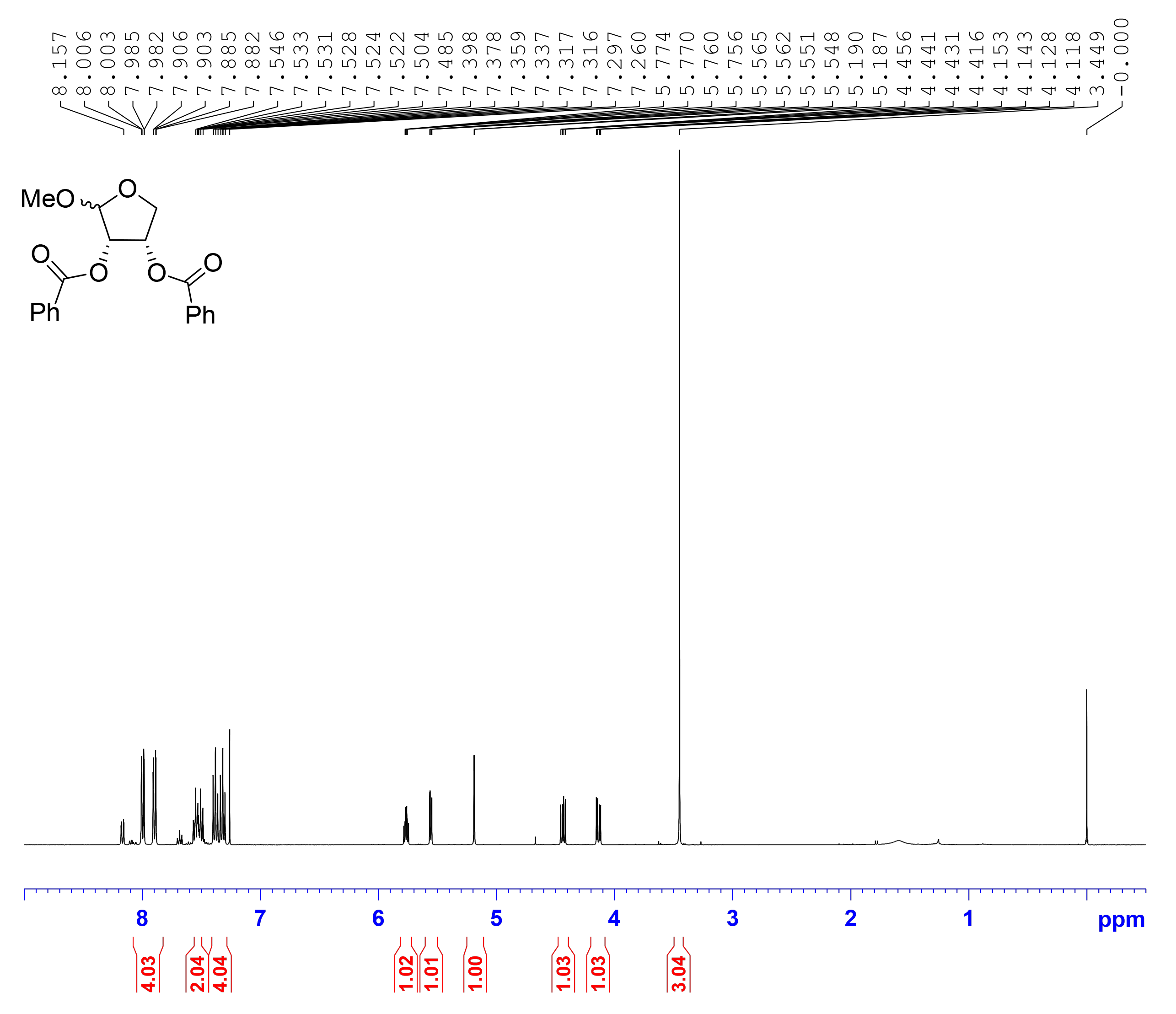

Supplement: Supplementary file 2 [file DataSheet1.ZIP › Supplementary Figure 4. 1H-NMR Methoxy-2-benzoic acid-3,4-tetrahydrofuran diester.tif]

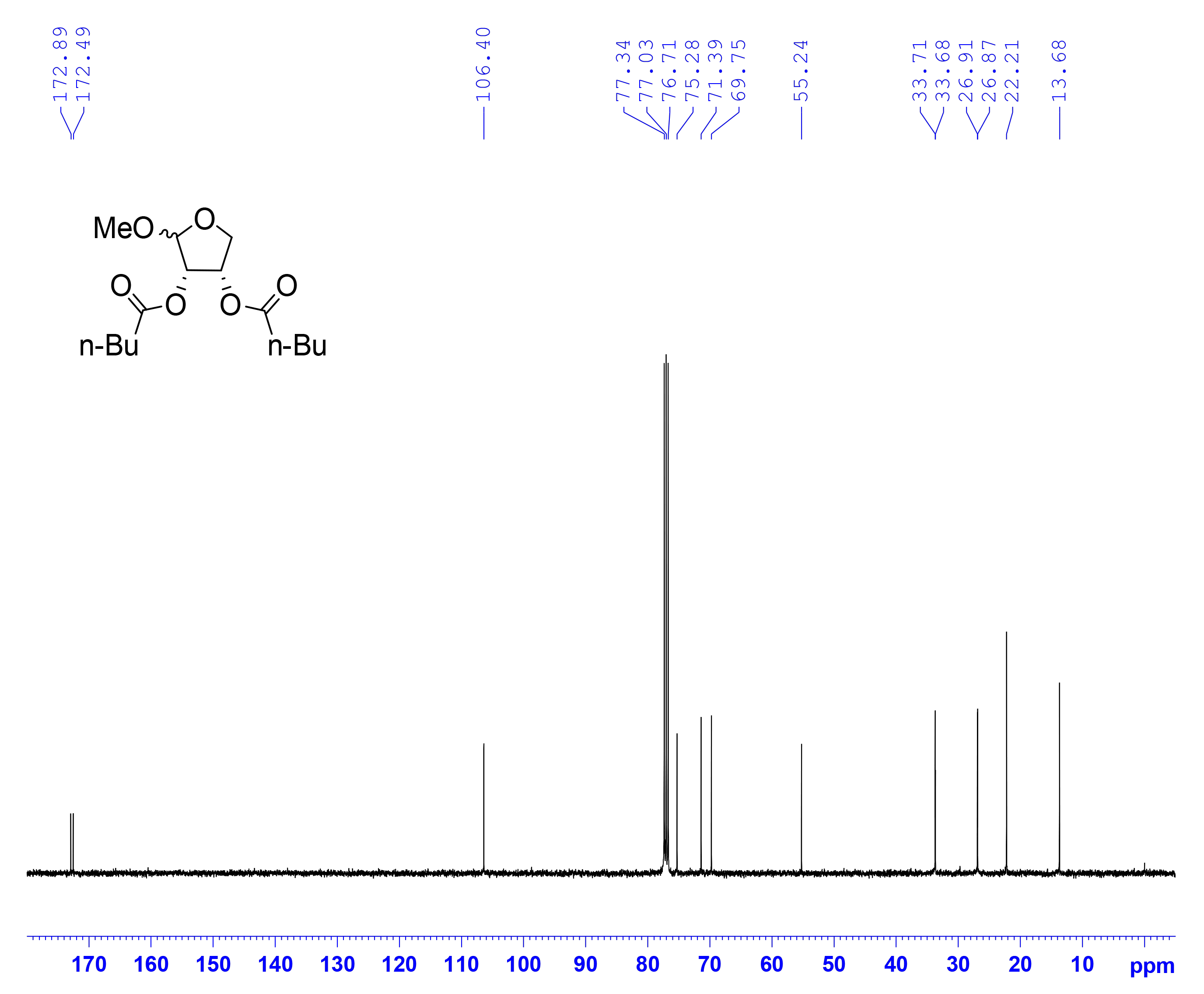

Supplement: Supplementary file 2 [file DataSheet1.ZIP › Supplementary Figure 5. 13C-NMR Methoxy-2-valeric acid-3,4-tetrahydrofuran diester.tif]

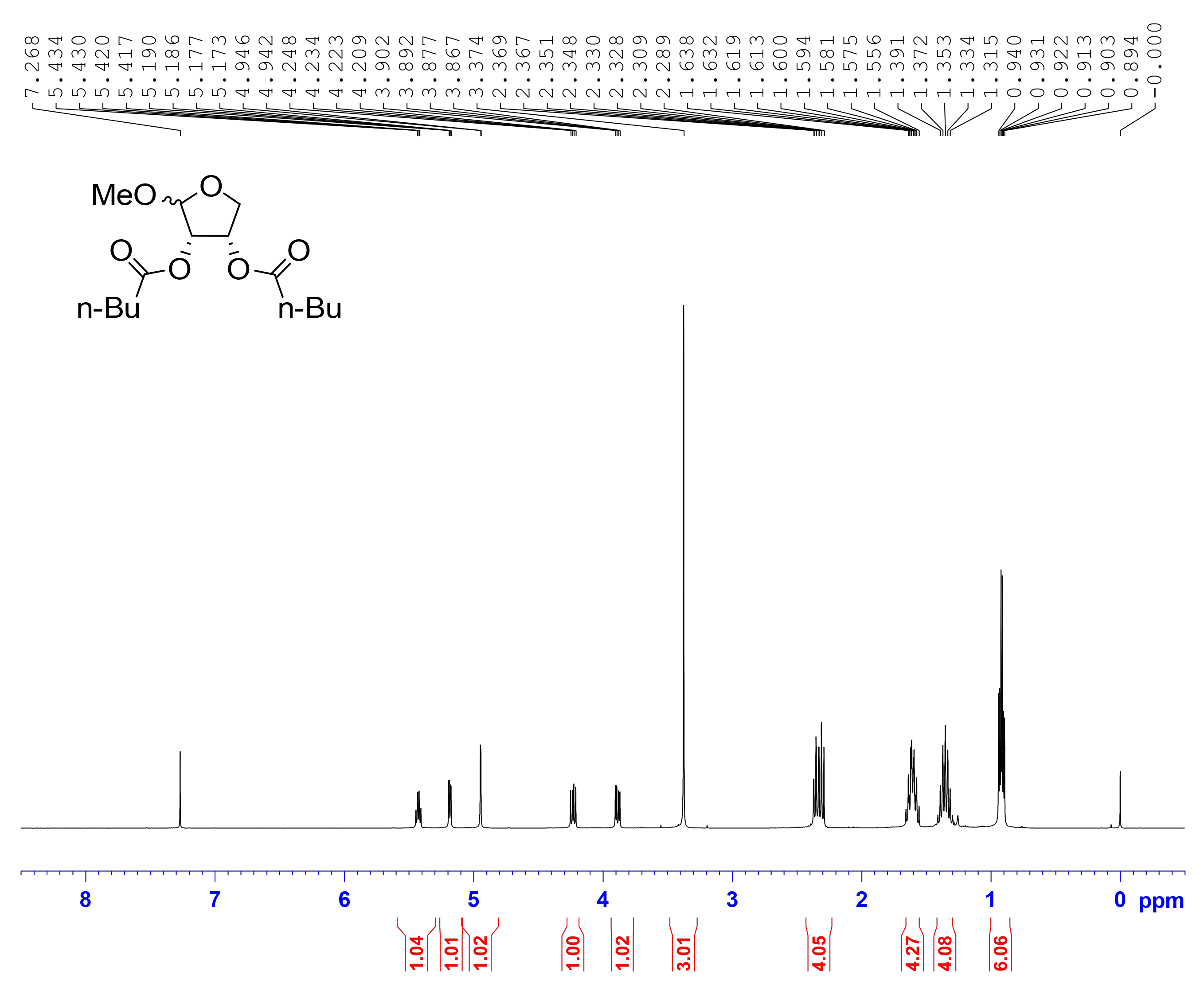

Supplement: Supplementary file 2 [file DataSheet1.ZIP › Supplementary Figure 6. 1H-NMR Methoxy-2-valeric acid-3,4-tetrahydrofuran diester.tif]

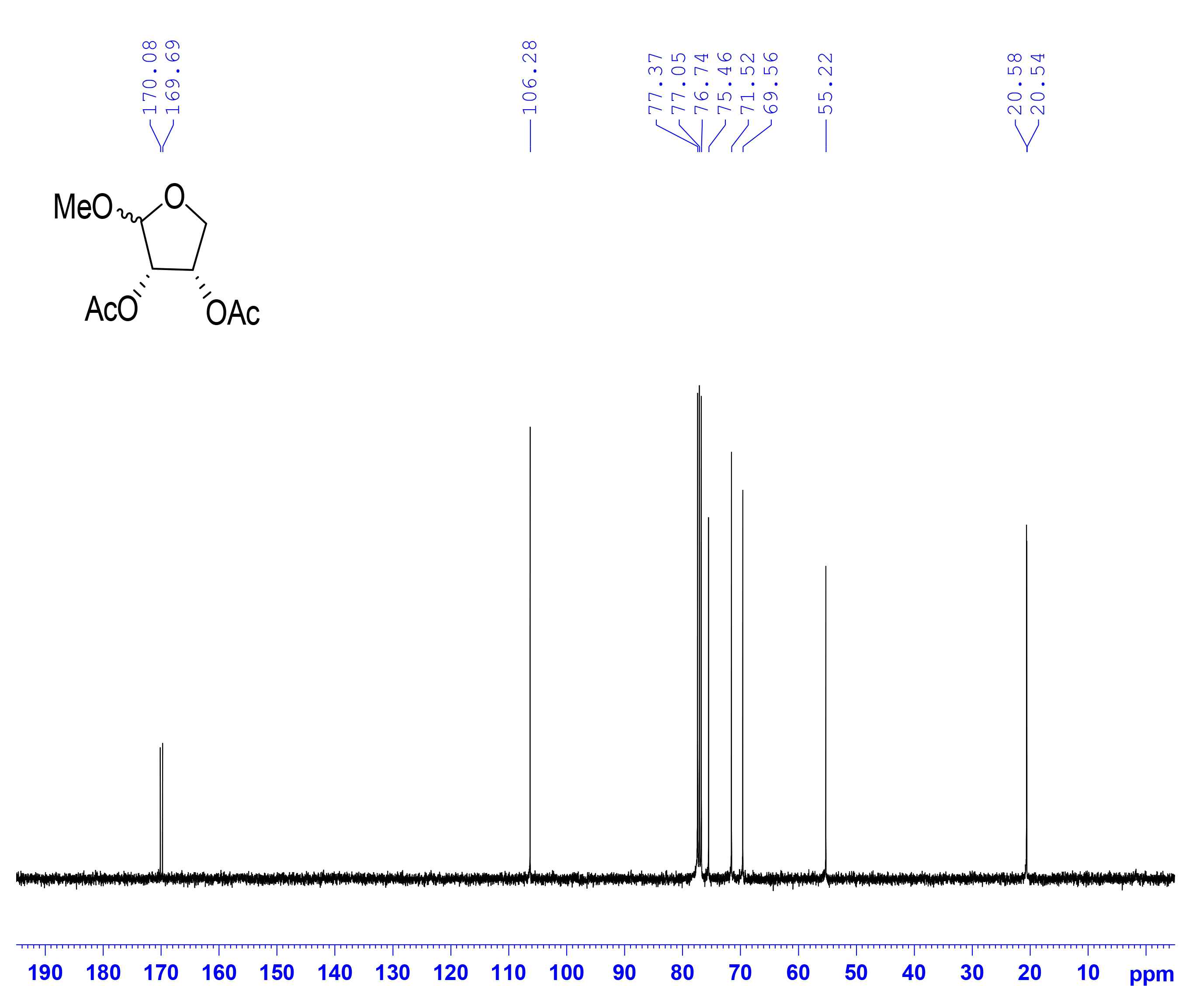

Supplement: Supplementary file 2 [file DataSheet1.ZIP › Supplementary Figure 7. 13C-NMR Methoxy-2-acetic acid-3,4-tetrahydrofuran diester.tif]

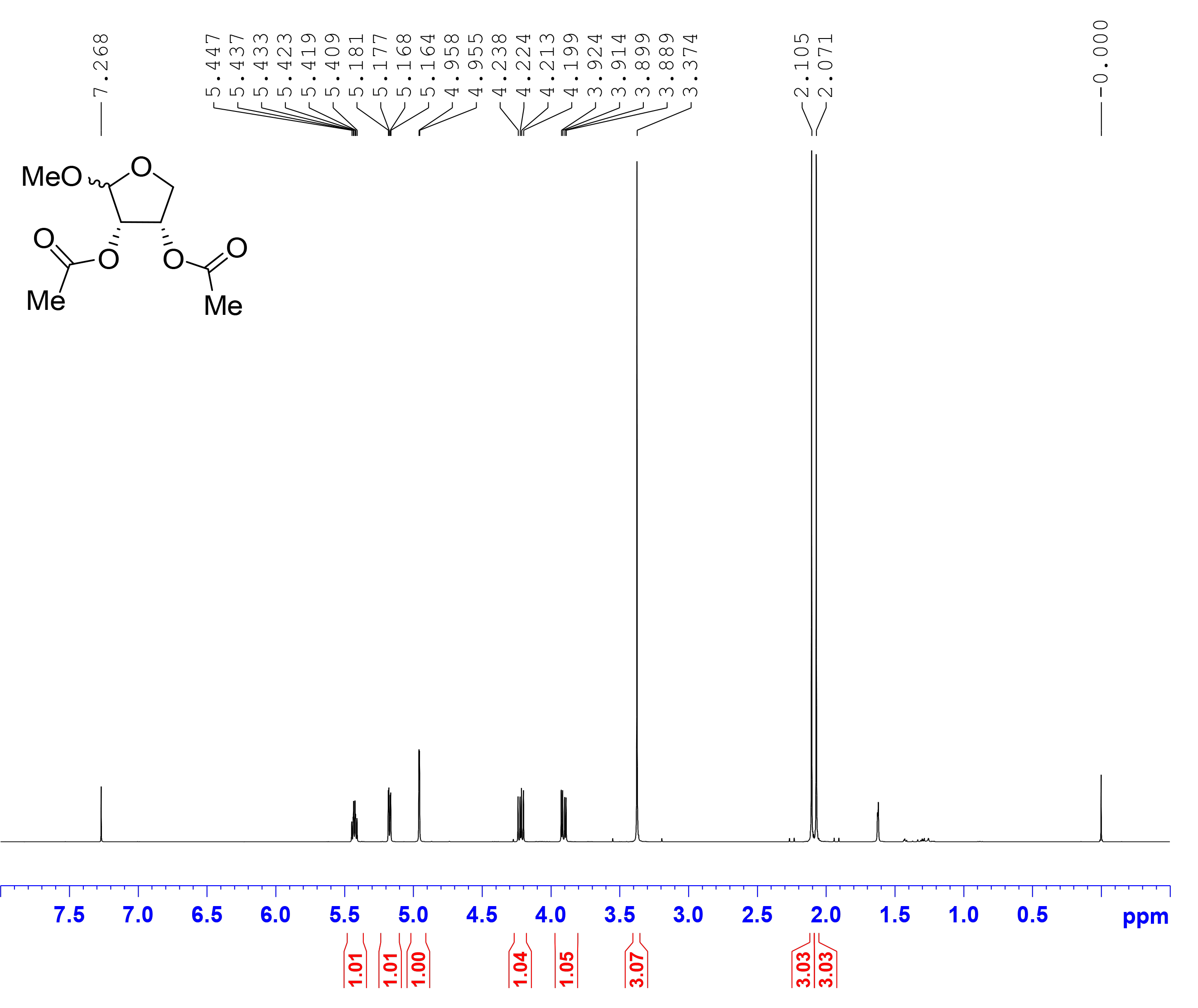

Supplement: Supplementary file 2 [file DataSheet1.ZIP › Supplementary Figure 8. 1H-NMR Methoxy-2-acetic acid-3,4-tetrahydrofuran diester.tif]

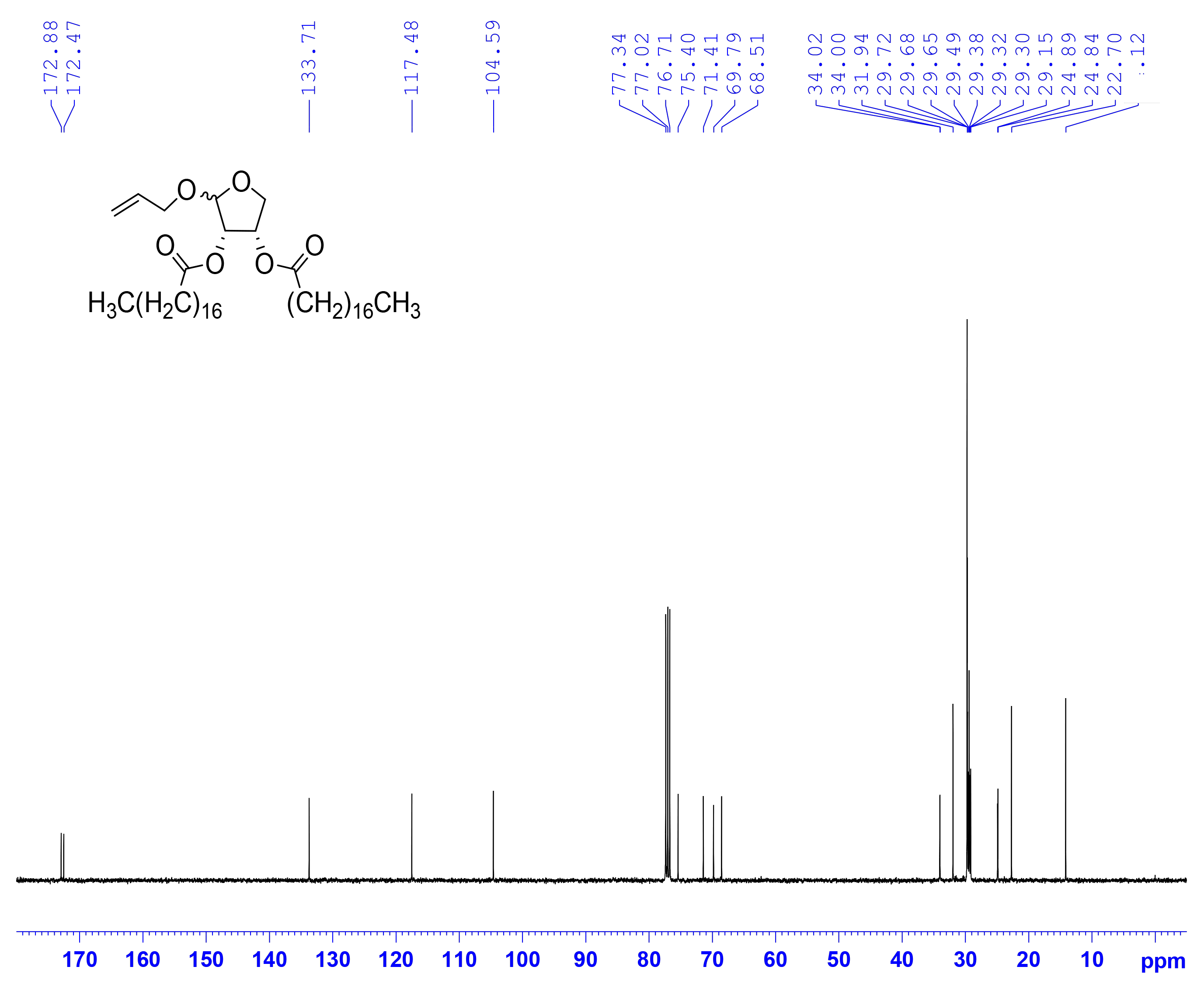

Supplement: Supplementary file 2 [file DataSheet1.ZIP › Supplementary Figure 9. 13C-NMR Allyloxy-2-octadecarbonate-3,4-tetrahydrofuran diester.tif]
